# A Slow Hydrogen Sulfide Donor GYY-4137 Partially Improves Vascular Function in Spontaneously Hypertensive Rats Fed a High-Fat Diet

**DOI:** 10.3390/pathophysiology32020027

**Published:** 2025-06-18

**Authors:** Basak G. Aydemir, Andrea Berenyiova, Martina Cebova, John D. Henderson, Andrej Barta, Sona Cacanyiova

**Affiliations:** 1 Institute of Normal and Pathological Physiology, Centre of Experimental Medicine, Slovak Academy of Sciences, 841 04 Bratislava, Slovakia; basak.aydemir@savba.sk (B.G.A.); andrea.berenyiova@savba.sk (A.B.); martina.cebova@savba.sk (M.C.); andrej.barta@savba.sk (A.Ba.); 2 Institute of Pathophysiology, Faculty of Medicine, Comenius University, 811 08 Bratislava, Slovakia; 3 Novo Nordisk Foundation Center for Basic Metabolic Research, University of Copenhagen, DK-2200 Copenhagen, Denmark; john.henderson@sund.ku.dk

**Keywords:** hydrogen sulfide, GYY-4137, high-fat diet, spontaneously hypertensive rat, vascular

## Abstract

Background/Objectives: Metabolic syndrome is one of the leading causes of mortality worldwide, with high-fat diet (HFD) intake being a significant driving force. Despite long-term research, new interventions are still being sought to improve cardiovascular disorders associated with metabolic syndrome. Methods: To explore the therapeutic potential of a slow-releasing H_2_S donor, we evaluated the effects of 3 weeks of treatment with GYY-4137 on systolic blood pressure (sBP), cardiac parameters, adiposity, selected plasma markers, and the vascular function of the thoracic aortas (TAs) and mesenteric arteries (MAs) isolated from male spontaneously hypertensive rats (SHRs) fed an HFD for 8 weeks. Results: HFD administration induced cardiac remodeling, increased adiposity, and decreased adrenergic contractility in both TAs and MAs. Moreover, although high-fat intake improved TAs relaxation, it decreased aortic protein expression of endothelial NO synthase and the involvement of NO in vasoactive responses of both TAs and MAs. In addition, protein expression of inducible NOS and tumor necrosis factor alpha (TNFα) in aortas was increased, as were plasma levels of chemerin, which has been proposed as a possible link among metabolic and vascular disorders and inflammation. Treatment with GYY-4137 reduced sBP, improved relaxation of the MAs, partially restored the contractility of the TAs, generally restored NO signaling, and decreased the protein expression of the inducible NOS and TNFα, as well as plasma chemerin levels. Conclusions: A slow H_2_S-releasing donor could partially ameliorate the metabolic changes induced by increased fat intake during essential hypertension and trigger beneficial vasoactive effects associated with the NO signaling restoration and suppression of inflammation.

## 1. Introduction

Characterized by a combination of symptoms, including insulin resistance, hypertension, and obesity, metabolic syndrome is one of the leading causes of mortality worldwide, and a high-fat diet (HFD) is the main driver. The global obesity epidemic directly contributes to cardiovascular disease risk factors, including dyslipidemia, type 2 diabetes, and hypertension. The WHO, in 2024, declared that overweight and obesity already affected a significant portion of the global population, with 43% being overweight and 16% being obese [1]. Metabolic syndrome initiated by chronic HFD consumption increases circulating levels of free fatty acids and inflammatory markers, causing chronic, systemic, low-grade inflammation and an unbalanced oxidative status in several organs [2]. New interventions that improve cardiovascular and metabolic health in these patients are essential to address the rising health problem that metabolic syndrome represents.

Targeting gasotransmitter signaling is a promising area of treatment for both cardiovascular and metabolic diseases. Hydrogen sulfide (H_2_S) is a gaseous transmitter that regulates intracellular signaling. The colonic microbiota represents the largest source of H_2_S in the body and results from recent years show that gut-derived H_2_S can induce systemic effects, and changes in H_2_S homeostasis in the colon H_2_S can be associated with various pathological stages, such as hypertension or atherosclerosis [3]. Many studies have been conducted on the effects of H_2_S produced enzymatically by various tissues of the cardiovascular system. However, the physiological and pathological role of H_2_S remains much less well understood than that of nitric oxide (NO), despite promising work from our group suggesting its potential antihypertensive function [4]. Research shows that strategies that increase the bioavailability of H_2_S are promising therapeutic approaches to treat diseases. Inorganic sulfide donors such as NaHS and Na_2_S provide a fast extracellular release of H_2_S and therefore have restricted therapeutic potential. One of the natural sources of H_2_S is polysulfides, which include garlic-derived compounds or isothiocyanates, including sulforaphane and erucin [5]. Synthetic H_2_S-donating compounds have also been developed. A very promising modern approach consists of designing multi-target molecular hybrids where some of the H_2_S-releasing moieties are combined with different drugs that are already used in the clinic to obtain novel multi-target molecular hybrids [6].

A slow-releasing H_2_S donor with vasodilatory and antihypertensive properties, GYY-4137, has been shown to be effective in models of cardiovascular disease [7]. GYY-4137 reduced vascular inflammation and oxidative stress, improved endothelial function, and reduced atherosclerotic plaque formation in apolipoprotein E−/− mice fed a high-fat diet [8]. Moreover, the long-term application of this donor led to a significant decrease in systolic blood pressure and alleviated myocardial fibrosis in spontaneously hypertensive rats (SHRs) [9]. In our recent work, we induced metabolic syndrome-like pathologies in SHRs via long-term fructose administration and reported that treatment with GYY-4137 alleviated fructose-induced structural malformations associated with tissue inflammation while also restoring the impaired vascular contractile ability [4]. In this study, we investigated the effects of GYY-4137 on blood pressure, biometric parameters, markers of inflammation, cardiac fibrosis, and both H_2_S and NO signaling in the vascular function of SHRs fed an HFD to provide further information on the therapeutic potential of an exogenous H_2_S donor in metabolic disorders. Another original contribution is the comparison of two types of arteries—the thoracic aorta (elastic type) and the mesenteric artery (muscular type)—and the evaluation of the importance of sulfide and nitroso pathways in the vasoactive responses of different arterial types. Similar observations have not been performed before. We hypothesized that metabolic syndrome-like pathological changes induced by HFD feeding in rats with essential hypertension would affect vascular function and the role of NO and H_2_S signaling pathways; however, these effects might be attenuated after GYY-4137 administration.

## 2. Materials and Methods

### 2.1. Ethics Statement

The animals were bred also in accordance with the European Convention for the Protection of Vertebrate Animals used for Experimental and other Scientific Purposes, Directive 2010/63/EU of the European Parliament. Animals were imported from the accredited breeding facility of the Center of Experimental Medicine, Slovak Academy of Sciences, Dobrá Voda, Slovak Republic, and were housed by the Institute of Normal and Pathological Physiology, Center of Experimental Medicine, Slovak Academy of Sciences (INPP CEM SAS). Poor health status was assessed daily using a point score for body, coat and skin condition, presence of discharge, presence of salivation and bite anomalies, impaired breathing, food and fluid intake, or sensory functions (impaired hearing or balance), and other atypical behavioral traits. The rats were free of infectious disease, were not immunodeficient, and were not genetically modified. An experimental protocol, including the research questions and analysis plan, was prepared before the study. Randomization was used to allocate rats to control and treatment groups using www.graphpad.com/quickcalcs/randomize1 (accessed on 2 November 2023). Animals were randomly measured in different orders; cages were rotated weekly to eliminate systematic influence of position. The experimenter was unaware of the animals’ group allocation during the outcome assessment. The first and corresponding authors were aware of the group allocation at the different stages of the experiment.

### 2.2. Experimental Animals

All the rats were maintained on a 12 h light/dark cycle in a temperature (20–22 °C)- and humidity (45–65%)-controlled room with access to standard laboratory rat chow (Altromin 1324P, Altromin Spezialfutter GmbH, Lage, Germany) or a high-fat obesity-inducing diet (Altromin, type C 1090-45, Altromin Spezialfutter GmbH, Lage, Germany) and access to drinking water ad libitum. The contents of the standard laboratory rat chow were 11% fat, 24% protein, and 65% carbohydrates, while the carbohydrate contents were as follows: 0 mg/kg monosaccharides, 47.8 mg/kg disaccharides, and 391.2 mg/kg polysaccharides. The remainder of the diet contained minerals and trace elements. The content of the high-fat diet was 45% fat, 18% protein, and 37% carbohydrates, and the carbohydrate content was 101.6 mg/kg monosaccharides, 52.1 mg/kg disaccharides, and 238.9 mg/kg polysaccharides; the remainder of the diet contained minerals and trace elements.

### 2.3. Experimental Design

An animal model of primary hypertension, SHRs, was used in this study. Twenty-four male SHRs aged 9 weeks and ranging in weight from 180 to 220 g were subsequently divided into three groups. One rat died during the systolic blood pressure measurements; thus, 23 SHRs were used for the experiments. The control SHR group (*n* = 8) received a standard diet for 8 weeks, the SHR + HFD group (*n* = 7) received a high-fat diet (HFD) for 8 weeks, and the SHR + HFD + GYY group (*n* = 8) received an HFD for 8 weeks. The SHR + HFD + GYY group also received GYY-4137 (266 µmol/kg/day dissolved in 1% dimethyl sulfoxide (DMSO) in physiological solution), which was injected intraperitoneally (i.p.) daily for three weeks at the age of 14–17 weeks. The rats in the SHR and SHR + HFD groups received vehicle via i.p. injection (1% DMSO in physiological solution; volumes were determined according to the actual body weight).

### 2.4. Blood Pressure Measurement and Biometric Parameters

Systolic blood pressure (sBP) was measured using tail-cuff plethysmography (MRBP; IITC Life Science Inc., Los Angeles, CA, USA). Animals were trained for the tail-cuff method through measuring blood pressure for two consecutive days before the evaluation of basal sBP levels (at the age of 9 weeks, the beginning of the HFD treatment). In all groups, sBP was subsequently measured at the end of the 14th week (the beginning of the treatment with GYY-4137) and at the end of the experiment (at the age of the 17 weeks). sBP was calculated as the average of the five measurements completed for each rat.

### 2.5. Body Weights and Diet Consumption

Rat body weight (BW) and food intake were monitored daily during the treatments. At the end of the experiment, the rats were euthanized by decapitation after brief CO_2_ anesthetization. CO_2_ was used to render the rats unconscious at a concentration of 30% to 40%, which was applied gradually to minimize stress and discomfort to the animal. Heart weight (HW), retroperitoneal adipose tissue weight (RTW), and the length of the tibia (TL) were measured. The ratios of HW to TL or BW and RTW to TL or BW were calculated to determine the degree of cardiac hypertrophy and body adiposity, respectively.

### 2.6. Blood Sample Analysis

Blood samples from the trunks of all the animals were collected at the end of the treatment day into preprepared heparinized tubes (140 UI/5 mL) and then centrifuged (850× *g*, 10 min, 4 °C, Centrifuge 5430 R, Eppendorf, Hamburg, Germany). The resulting plasma fraction was then analyzed to determine the levels of glucose (GLU), cholesterol (CHOL), triacylglycerol (TAG), urea, and creatine (CRE) using a biochemical analyzer and auxiliary reagent disks (Celercare, MNCHIP Technologies Co., Ltd., Tianjin, China). According to the manufacturer’s instructions for analysis, 100 μL of plasma was pipetted into the sample chamber and 430 μL of distilled water was then added to the diluent chamber of the test-specific reagent disk.

### 2.7. ELISA

Chemerin levels were measured in plasma samples using a commercially available Rat CHEM ELISA Kit (ELK Biotechnology, Denver, CO, USA) according to the manufacturer’s protocol. The samples of the trunk blood of the animals were collected at the end of the treatment into pre-prepared heparinized tubes (140 UI/5 mL) and were then centrifuged (850× *g*, 10 min, 4 °C) and the plasma samples were frozen immediately at −80 °C until the assay. After thawing on ice, serum samples were diluted 20-fold in the kit dilution buffer. A total of 100 μL of the diluted samples and the kit standards were then added to the ELISA plate and incubated at 37 °C for 80 min, after washing 3 times with 100 μL of wash buffer. A total of 100 μL of the biotinylated antibody working solution was added to each well and the plate was incubated at 37 °C for 50 min. The wells were then washed 3 times with 100 μL of wash buffer, followed by the addition of 100 μL of the Streptavidin-HRP working solution, and incubated at 37 °C for 50 min. The plate was then washed 5 times in the wash buffer, before 90 μL of TMB solution was added and the mixture was left in the dark for 20 min at 37 °C, after which 50 μL of the stop reagent was added. The plate was then read in a spectrophotometer at 450 nm.

### 2.8. Vasoactive Responses of Isolated Arteries

The descending part of the thoracic aorta (TA), beginning under the arcus aorta, and the superior mesenteric artery (MA) were isolated under a binocular microscope, cleaned of connective tissue and cut into 5 mm long rings with a preserved endothelium. The rings were set vertically between 2 stainless wire triangles and placed in a 20 mL incubation organ bath with Krebs solution (118 mmol/L NaCl; 5 mmol/L KCl; 25 mmol/L NaHCO_3_; 1.2 mmol/L MgSO_4_ × 7 H_2_O; 1.2 mmol/L KH_2_PO_4_; 2.5 mmol/L CaCl_2_; 11 mmol/L glucose; and 0.032 mmol/L CaNa_2_EDTA). The solution was oxygenated (95% O_2_ and 5% CO_2_) and maintained at 37 °C throughout the experiment.

Vasoactive changes in the TAs and MAs were determined using isometric tension sensors (FSG-01, MDE, Budapest, Hungary) connected to the upper triangle (the lower triangle was firmly fixed). The NI USB-6221 AD converter (MDE, Budapest, Hungary) and S.P.E.L. Advanced Kymograph software v. 5.10 (MDE, Budapest, Hungary) were used to record vascular changes.

Before the function of the arteries was tested, resting tension was applied to the rings, which was maintained for a stabilization period (45–60 min). A series of preliminary experiments was conducted to set the optimal value of resting tension. Different values of resting tension, from the lowest to the highest, were applied to rings of both arteries isolated from SHRs, and the rings were exposed to increasing concentrations of noradrenaline, which induced a concentration-dependent contractile response. The value of resting tension (1 g) at which the largest contractile response was repeatedly recorded was applied to the arterial rings used in this study. The arteries were then precontracted by adding noradrenaline (NA, 10^−6^ mol, Zentiva, Czech Republic) before a single dose of acetylcholine (Ach, 10^−5^ mol/L) was added, thus controlling the integrity of the endothelium and the contractile properties of smooth muscle cells. After 3 washes with physiological Krebs solution and an equilibration period, the experiment was started by applying cumulative doses of NA (10^−10^–10^−5^ mol/L) to determine the adrenergic contractions in TAs and MAs. Vasocontractile responses are presented in g as the measured changes in isometric tension. Cumulative doses of Ach (10^−10^–10^−5^ mol/L) were applied to NA-precontracted arteries to evaluate endothelium-dependent relaxation. The relaxation responses were determined as a percentage of the maximal NA-stimulated contraction. Individual inhibitors were used to analyze the involvement of the endogenous NO and H_2_S pathways in both contractile and vasorelaxant responses. Nonspecific inhibitors of NO synthase (NOS), NG-nitro-L-arginine methyl ester (L-NAME, 10^−4^ mol/L), or H_2_S-producing enzymes, such as cystathionine-γ-lyase (CSE) and DL-propargylglycine (PPG, 10 mmol/L), were added for 20 min to the organ bath, and the concentration-response curves for NA and Ach were generated again. Since we prepared two rings of the thoracic aorta and two rings of the mesenteric artery from each animal, individual inhibitors (LN and PPG) were not applied to the same ring. Areas under the curve (AUCs, in arbitrary units) were calculated and compared before and after incubation with the inhibitors to assess the involvement of NO and H_2_S in the vasoactive response in each group.

### 2.9. Analysis of Protein Expression

The protein expression levels of endothelial and inducible NOS isoforms, cystathionine γ-lyase (CSE), cystathionine β-synthase (CBS), and tumor necrosis factor alpha (TNFα), which are inflammatory factors, in the abdominal aorta and transforming growth factor-beta (TGF-β) in the left ventricle were determined via Western blot analysis. Briefly, aortic tissue lysates were prepared by homogenization in protease inhibitor-supplemented 0.05 mM Tris buffer (protease inhibitor cocktail), followed by centrifugation and a measurement of the protein concentration using the Lowry assay. The supernatants were then subjected to SDS-PAGE on 12% or 15% gels for protein separation and transferred to nitrocellulose membranes. Immunoblotting was performed using primary antibodies against endothelial eNOS (1:1000 dilution) (Abcam, Cambridge, UK), inducible iNOS (1:1000 dilution), CSE (1:5000 dilution), CBS (1:3000 dilution), and TNF-α (1:3000 dilution) (Proteintech, Manchester, UK), and β-actin (1:5000 dilution) (Abcam, Cambridge, UK) was used as a loading control. Primary antibodies were incubated at 4 °C overnight. The membrane was incubated with secondary peroxidase-conjugated antibodies; eNOS (1:5000 dilution), iNOS (1:5000 dilution), CBS (1:5000 dilution), β-actin (1:5000 dilution) anti-rabbit (Abcam, Cambridge, UK), CSE (1:3000 dilution), TNF-α (1:3000 dilution) anti-mouse (Cell Signaling, Danvers, MA, USA), anti-TGF β (1:1000 dilution), anti-rabbit, and polyclonal (Sigma-Aldrich, Germany) at room temperature for 2 h, followed by enhanced chemiluminescence (ECL) reagent (BioRad, Inc., Hercules, CA, USA), which facilitated band visualization and subsequent quantification using standardized software (Image LabTM Touch software 2.4 BioRad, Inc., Hercules, CA, USA). The results were normalized to β-actin expression for accurate representation.

### 2.10. Total NO Synthase Activity

NOS activity was determined in the aortic arch and tissue remnants from the thoracic aorta. The isolated aortic tissue samples were homogenized, and nitric oxide synthase (NOS) activity was quantified by measuring the conversion of [3H]-L-arginine (obtained from MP Biochemicals) to [3H]-L-citrulline, according to a previously established method [10]. [3H]-L-citrulline levels were measured using the Quanta Smart TriCarb Liquid Scintillation Analyzer (TriCarb, Packard, UK). NOS activity was then normalized to the protein content and reported as picokatal per gram of protein (pkat/g protein).

### 2.11. Collagen Amount

The amount of collagen in tissue was quantified by measuring hydroxyproline (hyp), an amino acid that is a major component of collagen. To determine the amount of hyp in the left ventricle, we used a modified method developed by to Reddy and Enwemeka [11]. Briefly, we used the same homogenate as used for the measurement of NOS activity. A total of 25 µL of the sample was placed into a test tube and subjected to hydrolysis by 50 µL of 2 mol/L NaOH. The samples were then heated to 110 °C for 60 min, which breaks down the tissue and converts the collagen’s hydroxyproline into a free form that can be measured. After hydrolysis, the tissue samples were neutralized with 450 µL of chloramine-T for 25 min in room temperature. Following chloramine-T treatment, 500 µL of p-dimethylaminobenzaldehyde (DMAB) was added to the sample. Incubation lasted 20 min at 65 °C. The absorbance of the resulting solution was measured at 550 nm using a spectrophotometer. The collagen content in the tissue samples was calculated using a hydroxyproline standard calibration curve. The samples were repeated in triplicate to ensure consistency in the results. It was assumed that 12.5% of the collagen is composed of hydroxyproline. A standard curve was prepared by creating a series of hydroxyproline standards with known concentrations.

### 2.12. Statistical Analysis

The data are presented as the means ± S.E.Ms. The group size was calculated via a priori analysis via G*Power software v3.1. The normality of data distribution was verified by the Shapiro–Wilk test. Homogeneity of variances (equality of variances) was verified by the Levene test. For the statistical comparison of sBP and vasoactive responses between groups, two-way analysis of variance (ANOVA) and the Bonferroni post hoc correction were used, or paired Student’s *t* tests (AUCs before and after inhibitor treatments). One-way ANOVA was used to evaluate general cardiovascular parameters, plasma parameters, NO synthase activity, collagen content, and protein expression. Differences in means between groups were considered significant at *p* < 0.05. The data were analyzed with OriginPro 2019b (OriginLab Corporation, Northampton, MA, USA).

### 2.13. Drugs

All the chemicals used in this study were purchased from Merck Life Science Ltd., Bratislava, Slovakia, unless stated otherwise.

## 3. Results

### 3.1. General Parameters of the Experimental Animals

A total of 23 rats (8 in the SHR group, 7 in the SHR + HFD group, and 8 in the SHR + HFD + GYY group, Table 1) were included in the study and divided into three groups according to whether they received the HFD and were treated with the H_2_S donor GYY-4137. At the beginning of the experiment, no difference in basal body weight (BW) was observed between the groups: SHR—244.12 ± 6.98 g, SHR + HFD—238.50 ± 4.90 g, and SHR + HFD + GYY—230.13 ± 3.68 g. From the beginning to the end of the experiment, we noticed a significant increase in BW in all groups (F_(2,65)_ = 83.14; *p* = 9.25 × 10^−18^), and the greatest change was observed in the HFD group. The HFD induced significant increases in BW, retroperitoneal adipose tissue weight (RTW), and tibia length (TL). Additionally, chronic consumption of the HFD increased the ratios of RTW to BW (RTW/BW) and RTW to TL (RTW/TL) (Table 1). These results confirmed increased adiposity in the HFD group. However, treatment with GYY-4137 did not affect these parameters.

Neither the HFD nor treatment with GYY-4137 significantly altered the plasma glucose (GLU) levels. A significant increase in the plasma triacylglycerol (TAG) levels was observed in the HFD group, as expected. However, the plasma cholesterol (CHOL) level was lower in the HFD group than in the SHR group. The urea level and the ratio of urea to creatinine (Urea/CRE) were significantly altered in the groups that received the HFD compared with those in the control diet (SHR) group; however, treatment with GYY-4137 partially attenuated this effect. HFD intake significantly increased chemerin levels in plasma; on the other hand, GYY-4137 administration returned the level of chemerin to control levels. Food intake was significantly lower in the HFD group than in the control diet (SHR) group, and this effect was increased through treatment with GYY-4137 (Table 1).

### 3.2. Cardiac Parameters of the Experimental Animals

Chronic consumption of the HFD increased heart weight (HW) as well as the ratios of HW to BW and HW to TL, suggesting the occurrence of cardiac hypertrophy. Moreover, HFD increased the protein expression of the central regulator in the development of fibrosis TGF-β, as well as collagen content in the left ventricle. The treatment with GYY-4137 returned TGF-β to control levels, and collagen levels decreased, but not significantly (Table 2).

### 3.3. Blood Pressure of the Experimental Animals

**Figure 1:**
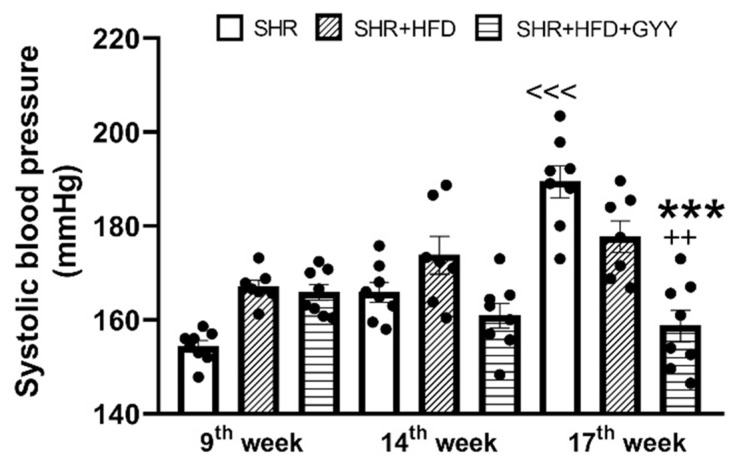
The systolic blood pressure of rats. The following groups were used: the control SHR (SHR, *n* = 8), SHR receiving HFD (SHR + HFD, *n* = 7), and SHR receiving HFD and 266 µmol/kg/day GYY-4137 (SHR + HFD + GYY, *n* = 8) groups. The data are shown as the means ± SEMs. The statistical analysis was performed by two-way ANOVA with the Bonferroni post hoc correction. N—number of rats. *** *p* < 0.001 compared with the SHR group (17th week), ^++^ *p* < 0.01 compared with the SHR + HFD group (17th week), <<< *p* < 0.001 compared with the SHR group (14th week).

sBP measurements for all groups were recorded at week 9, week 14 (after 5 weeks of HFD consumption), and week 17 (after 8 weeks of HFD consumption and 3 weeks of GYY-4137 treatment). Two-way ANOVA confirmed that sBP differed not only depending on the administration of a high-fat diet or GYY (F_(2,61)_ = 12.81; *p* ˂ 0.0001) but also depending on time (F_(2,61)_ = 14.62; *p* ˂ 0.0001). Although the Bonferroni post hoc test only confirmed a significant increase in sBP over time in the control SHR group (*p* ˂ 0.001), at the end of the experiment, a significant reduction in sBP was observed in the group treated with GYY-4137 compared with both the SHR and HFD groups (*p* < 0.001 and *p* < 0.01) (Figure 1).

### 3.4. Endothelial Function and Contractility of the Thoracic Aorta and Mesenteric Artery

**Figure 2:**
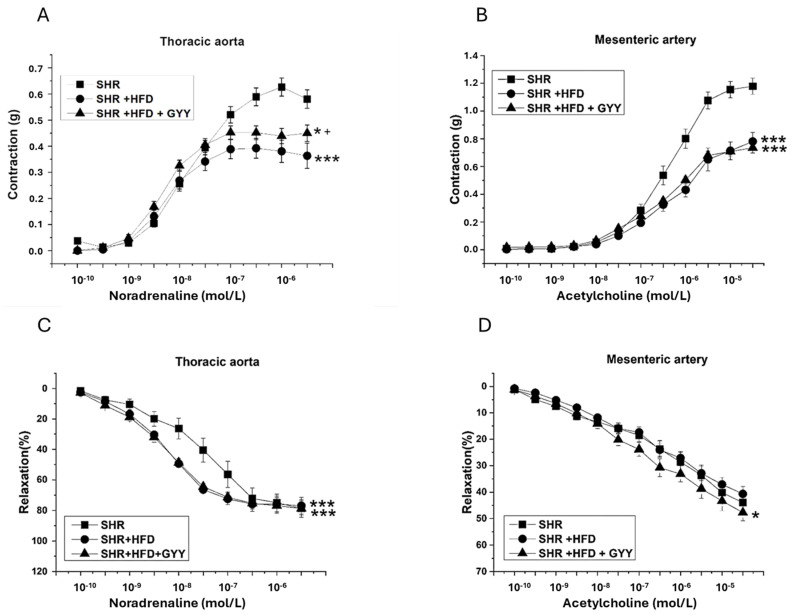
Contractile responses induced by noradrenaline in the thoracic aorta (**A**) and mesenteric artery (**B**) and endothelium-dependent vasorelaxant responses induced by acetylcholine in the thoracic aorta (**C**) and mesenteric artery (**D**). Arteries were isolated from control rats (SHRs), SHRs fed the HFD (SHR + HFD), and SHRs fed the HFD and treated with GYY-4137 (SHR + HFD + GYY). Number of rats: thoracic aorta—SHR group *n* = 6, SHR + HFD group *n* = 6–7, and SHR + HFD + GYY group *n* = 7–8; and mesenteric artery—SHR group *n* = 7, SHR + HFD group *n* = 6, and SHR + HFD + GYY group *n* = 6–7. The results are presented as the means ± S.E.Ms. Statistical analyses were performed by two-way ANOVA with the Bonferroni post hoc correction. * *p* < 0.05, and *** *p* < 0.001 compared with the SHR group, and ^+^ *p* < 0.05 compared with the SHR + HFD group.

Contractile responses were induced through the activation of adrenergic receptors through the successive application of exogenous noradrenaline (NA) at concentrations ranging from 10^−10^ to 3 × 10^−6^ mol/L (TA) or 3 × 10^−5^ mol/L (MA). HFD intake induced a significant decrease in NA-induced contraction of both the TA (F_(2,218)_ = 12.71; *p* = 6.59 × 10^−6^) (Figure 2A) and MA (F_(2,238)_ = 70.20; *p* < 6.35 × 10^−24^) (Figure 2B), which indicates deterioration of the contractile properties. On the other hand, the administration of GYY-4137 partially improved the inhibited contractile response in the TA only (*p* < 0.05, Figure 2A). Endothelial function was subsequently investigated by applying cumulative doses of acetylcholine (10^−10^ to 3 × 10^−6^ mol/L in the TA or 3 × 10^−5^ mol/L in the MA) to the NA-precontracted arterial rings. Compared with the SHR group, both the HFD and the GYY-4137 treatment significantly increased endothelium-dependent vasorelaxation in the TA, although the maximum response remained unchanged (F_(2,208)_ = 14.03; *p* = 2.17 × 10^−6^) (Figure 2C). The vasorelaxant response of the MA remained unaffected by the HFD, but following treatment with GYY-4137, a significant increase was observed (F_(2,238)_ = 8.52; *p* = 2.79 × 10^−4^) (Figure 2D). The maximum reached responses and molar concentrations of noradrenaline and acetylcholine that induced the half-maximum response (EC50) are shown in Table 3 (thoracic aorta) and Table 4 (mesenteric artery).

### 3.5. Evaluation of NO/NOS System Participation

**Figure 3:**
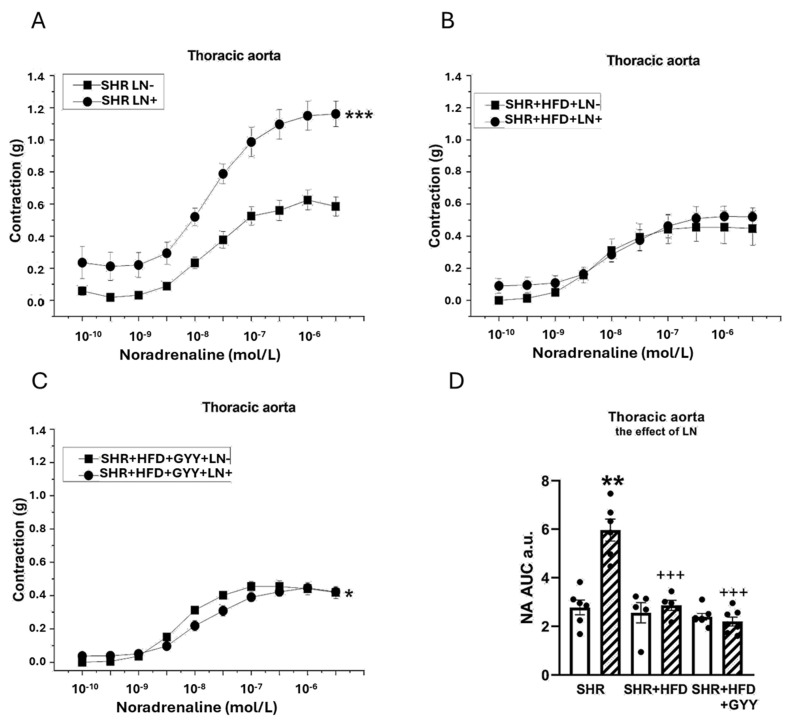
The effects of N^G^-nitro-L-arginine methyl ester (LN) on the contractile responses of the thoracic aorta induced by noradrenaline in control SHRs (**A**), SHRs fed the HFD (**B**), and SHRs fed the HFD and treated with GYY-4137 (**C**). The level of NO participation was determined via an AUC evaluation (**D**). White column—before the addition of the inhibitor; hatched column—after the addition of the inhibitor. The number of arterial segments per group was as follows: SHR *n* = 6; SHR + HFD *n* = 5; and SHR + HFD + GYY *n* = 7. The results are presented as the means ± S.E.Ms. The statistical analysis was performed by two-way ANOVA (line graphs) or paired Student’s *t* test/one-way ANOVA (AUC) with the Bonferroni post hoc correction. * *p* < 0.05, ** *p* < 0.01, *** *p* < 0.001 vs. LN within the relevant group; ^+++^ *p* < 0.05 vs. SHR LN+.

**Figure 4:**
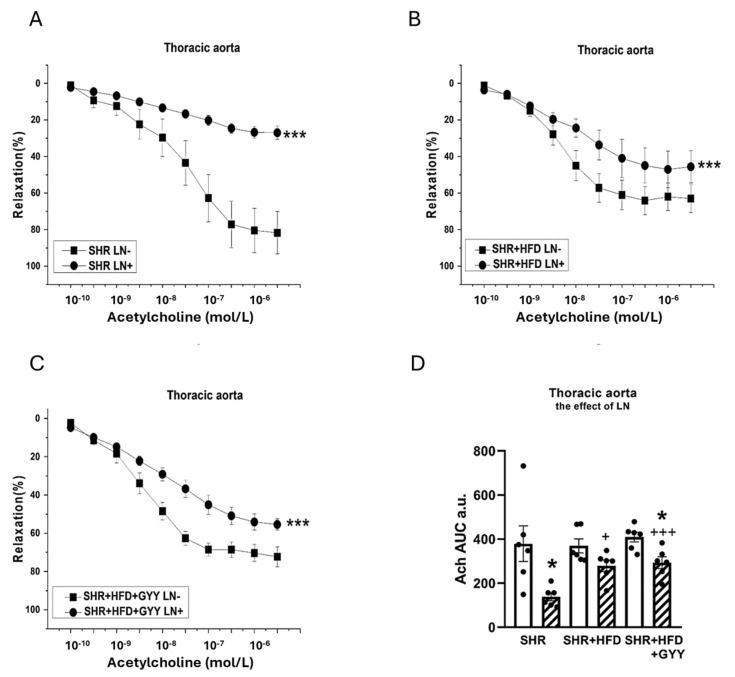
Effects of N^G^-nitro-L-arginine methyl ester (LN) on the relaxation responses of the thoracic aorta induced by acetylcholine in control SHRs (**A**), SHRs fed the HFD (**B**), and SHRs fed the HFD and treated with GYY-4137 (**C**). The level of NO participation was determined via an AUC evaluation (**D**). White column—before the addition of the inhibitor; hatched column—after the addition of the inhibitor. The number of arterial segments per group was as follows: SHR *n* = 6; SHR + HFD *n* = 6; and SHR + HFD + GYY *n* = 6. The results are presented as the means ± S.E.Ms. The statistical analysis was performed by two-way ANOVA (line graphs) or paired Student’s *t* test/one-way ANOVA (AUC) with the Bonferroni post hoc correction. * *p* < 0.05, *** *p* < 0.001 vs. LN within the relevant group; ^+^ *p* < 0.05; ^+++^ *p* < 0.001 vs. SHR LN+.

**Figure 5:**
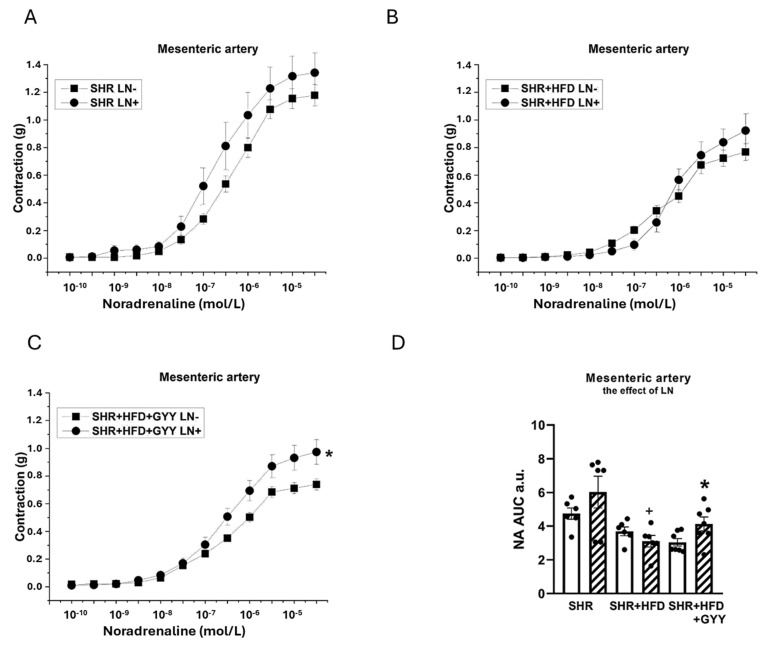
Effects of N^G^-nitro-L-arginine methyl ester (LN) on the contractile responses of the mesenteric artery induced by noradrenaline in control SHRs (**A**), SHRs fed the HFD (**B**), and SHRs fed the HFD and treated with GYY-4137 (**C**). The level of NO participation was determined via an AUC evaluation (**D**). White column—before the addition of the inhibitor; hatched column—after the addition of the inhibitor. The number of arterial segments per group was as follows: SHR *n* = 6; SHR + HFD *n* = 6; and SHR + HFD + GYY *n* = 7. The results are presented as the means ± S.E.Ms. The statistical analysis was performed by two-way ANOVA (line graphs) or a paired Student’s *t* test/one-way ANOVA (AUC) with the Bonferroni post hoc correction. * *p* < 0.05 vs. LN within the relevant group; ^+^ *p* < 0.05 vs. SHR LN+.

**Figure 6:**
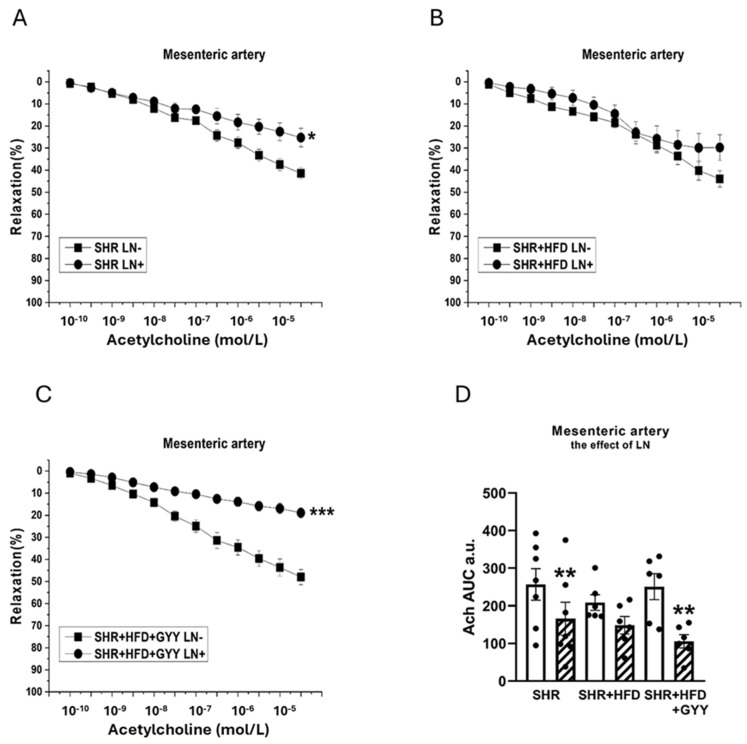
Effects of N^G^-nitro-L-arginine methyl ester (LN) on relaxation responses of the mesenteric artery induced by acetylcholine in control SHRs (**A**), SHRs fed the HFD (**B**), and SHRs fed the HFD and treated with GYY-4137 (**C**). The level of NO participation was determined via an AUC evaluation (**D**). White column—before the addition of the inhibitor; hatched column—after the addition of the inhibitor. The number of arterial segments per group was as follows: SHR *n* = 7; SHR + HFD *n* = 6; and SHR + HFD + GYY *n* = 6. The results are presented as the means ± S.E.Ms. The statistical analysis was performed by two-way ANOVA (line graphs) or a paired Student’s *t* test/one-way ANOVA (AUC) with the Bonferroni post hoc correction. * *p* < 0.05, ** *p* < 0.01, and *** *p* < 0.001 compared with the relevant group without LN treatment.

The arterial rings were incubated with N^G^-nitro-L-arginine methyl ester (LN; 10^−4^ mol/L) for 20 min to analyze the participation of NO signaling in vasoactive responses. The NA-induced contractile response of the TA of the SHR group was significantly increased after treatment with LN (F_(1,118)_ = 147; *p* < 0.0001; Figure 3A). However, treatment with LN had no effect on the contractile response of the SHR + HFD group (F_(1,78)_ = 1.98; *p* = 0.16) (Figure 3B). The contractile response of the TA of the SHR + HFD + GYY group was further significantly increased after treatment with LN (F_(1,138)_ = 5.08; *p* = 0.03; Figure 3C). The results of the AUC evaluation revealed that after the LN incubation, the contractile response of the TAs was significantly increased in the control group (*p* < 0.01); however, both HFD consumption and treatment with GYY-4137 reduced the influence of NO (both *p* < 0.001, Figure 3D). In terms of endothelial function, treatment with LN had a significant effect on acetylcholine-induced relaxation in the TA, and the vasorelaxation responses were significantly reduced in the SHR (F_(1,118)_ = 69.7; *p* = 4.03 × 10^−13^), SHR + HFD (F_(1,58)_ = 17.1; *p* = 1.74 × 10^−4^), and SHR + HFD + GYY (F_(1,118)_ = 63.1; *p* = 2.96 × 10^−12^) groups (Figure 4A–C). The AUC analysis revealed that the HFD decreased the participation of NO in the relaxation response of TAs, which was restored after treatment with GYY-4137 (*p* < 0.05, Figure 4D). In the MA, in contrast to the control SHR group and HFD group, only the chronic administration of GYY-4137 significantly increased the participation of NO in the contractile response induced by NA (*p* < 0.05, Figure 5A–D). With respect to endothelial function, we observed a significant reduction in the relaxation response in the MA of the SHR control (*p* < 0.05) and GYY-4137 groups (*p* < 0.001) after LN treatment (Figure 6A,D), which was confirmed by the AUC analysis (both *p* < 0.01, Figure 6D).

### 3.6. Evaluation of H_2_S/CSE System Participation

Acute treatment with the specific CSE inhibitor DL-propargylglycine (PPG, 10 mmoL/L) was used to analyze the contribution of H_2_S to both relaxant and contractile responses. The direct application of PPG had distinct effects on the TA and MA. PPG administration to the TA evoked a slight increase in basal tone (0.127 ± 0.04 g). This effect was reversed by HFD consumption (−0.145 ± 0.041 g; *p* < 0.001 vs. the SHR group), but GYY administration returned it to control levels (0.043 ± 0.051 g; *p* < 0.05 vs. the HFD group). PPG application in MA hardly affected the basal tone, indicating minimal basal production of H_2_S (0.033 ± 0.026 g). The consumption of the HFD induced a slight decrease in basal tone (−0.113 ± 0.026; *p* < 0.001 vs. the SHR group), which was partially alleviated by the administration of GYY (−0.032 ± 0.018 g; *p* < 0.05 vs. the HFD group; *p* < 0.01 vs. the SHR group).

**Figure 7:**
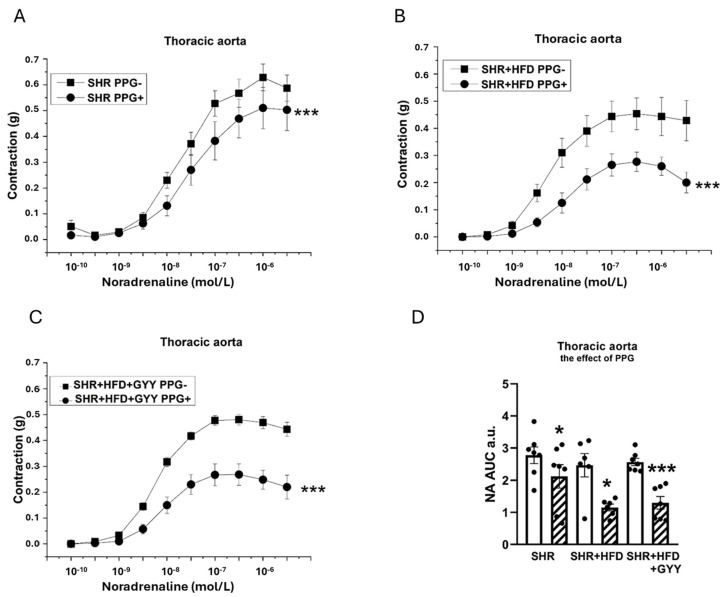
Effect of DL-propargylglycine (PPG) on the contractile response of the thoracic aorta induced by noradrenaline in control SHRs (**A**), SHRs fed the HFD (**B**), and SHRs fed the HFD and treated with GYY-4137 (**C**). The level of H_2_S participation was evaluated via the AUC (**D**). White column—before the addition of the inhibitor; hatched column—after the addition of the inhibitor. The number of arterial segments per group was as follows: SHR *n* = 7; SHR + HFD *n* = 6; and SHR + HFD + GYY *n* = 7. The results are presented as the means ± S.E.Ms. The statistical analysis was performed by two-way ANOVA (line graphs) or paired Student’s *t* test/one-way ANOVA (AUC) with the Bonferroni post hoc correction. * *p* < 0.05 and *** *p* < 0.001 compared within the relevant group without PPG treatment.

**Figure 8:**
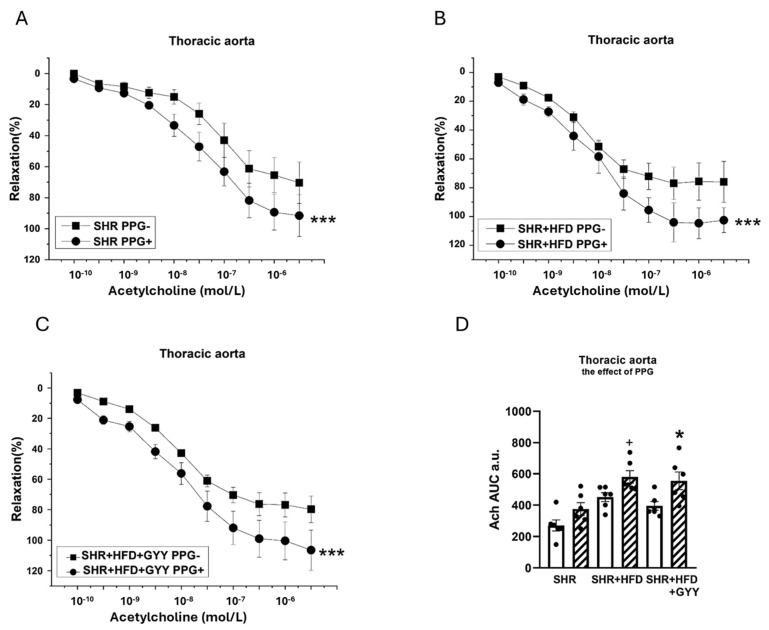
Effects of DL-propargylglycine (PPG) on the relaxation responses of the thoracic aorta induced by acetylcholine in control SHRs (**A**), SHRs fed the HFD (**B**), and SHRs fed the HFD and treated with GYY-4137 (**C**). The level of H_2_S participation was evaluated via the AUC (**D**). White column—before the addition of the inhibitor; hatched column—after the addition of the inhibitor. The number of arterial segments per group was as follows: SHR *n* = 6; SHR + HFD *n* = 6; and SHR + HFD + GYY *n* = 6. The results are presented as the means ± S.E.Ms. The statistical analysis was performed by two-way ANOVA (line graphs) or paired Student’s *t* test/one-way ANOVA (AUC) with the Bonferroni post hoc correction. * *p* < 0.05 and *** *p* < 0.001 compared within the relevant group without PPG treatment; ^+^ *p* < 0.05 compared with the SHR + PPG group.

**Figure 9:**
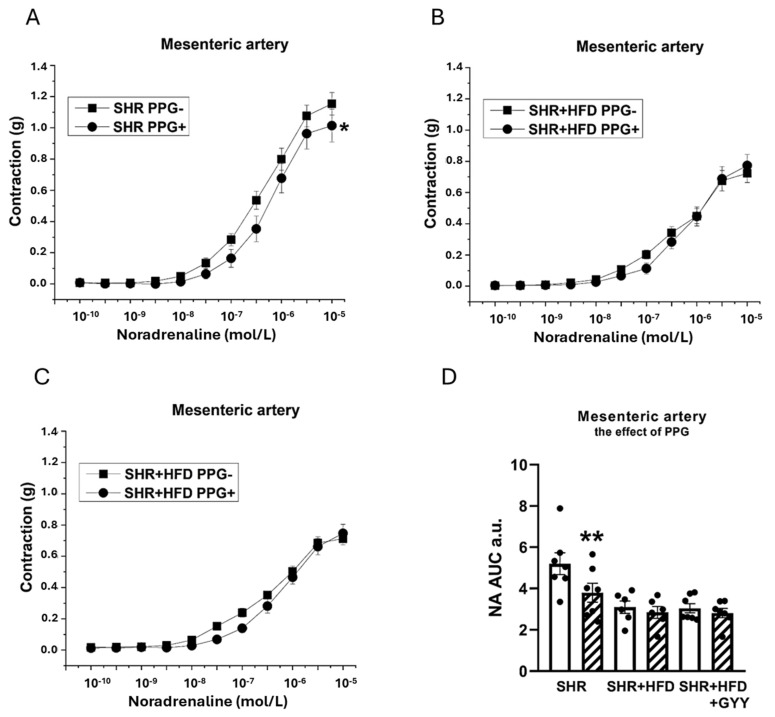
Effect of DL-propargylglycine (PPG) on the contractile response of the mesenteric artery induced by noradrenaline in control SHRs (**A**), SHRs fed the HFD (**B**), and SHRs fed the HFD and treated with GYY-4137 (**C**). The level of H_2_S participation was evaluated via the AUC (**D**). White column—before the addition of the inhibitor; hatched column—after the addition of the inhibitor. The number of arterial segments per group was as follows: SHR *n* = 7; SHR + HFD *n* = 6; and SHR + HFD + GYY *n* = 7. The results are presented as the means ± S.E.Ms. The statistical analysis was performed by two-way ANOVA (line graphs) or paired Student’s *t* test/one-way ANOVA (AUC) with the Bonferroni post hoc correction. * *p* < 0.05 and ** *p* < 0.01 compared with the relevant group without PPG treatment.

**Figure 10:**
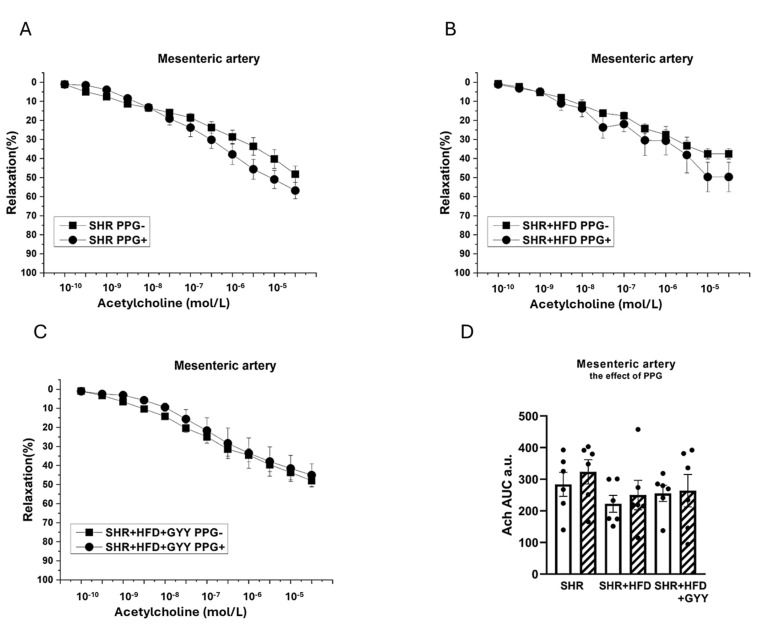
Effects of DL-propargylglycine (PPG) on the relaxation responses of the thoracic aorta induced by acetylcholine in control SHRs (**A**), SHRs fed the HFD (**B**), and SHRs fed the HFD and treated with GYY-4137 (**C**). The level of H_2_S participation was evaluated via the AUC (**D**). White column—before the addition of the inhibitor; hatched column—after the addition of the inhibitor. The number of arterial segments per group was as follows: SHR *n* = 6; SHR + HFD *n* = 6; and SHR + HFD + GYY *n* = 6. The results are presented as the means ± S.E.Ms. The statistical analysis was performed by two-way ANOVA (line graphs) or paired Student’s *t* test/one-way ANOVA (AUC) with Bonferroni post hoc test.

Regarding the contractions induced by NA, the inhibitor was applied to the organ bath 20 min before the implementation of concentration-dependent responses. The TA exhibited significantly reduced contractile responses after treatment with PPG in the SHR (F_(1,138)_ = 11.5; *p* = 9.28 × 10^−4^), SHR + HFD (F_(1,118)_ = 46.1; *p* = 8.27 × 10^−10^), and SHR + HFD + GYY (F_(1,157)_ = 120.3; *p* < 0.0001) groups (Figure 7A–C). The levels of H_2_S participation determined by calculating the AUC revealed that endogenously produced H_2_S stimulated a pro-contractile effect, and this effect on the TA was not changed by either HFD consumption or treatment with GYY-4137 (Figure 7D). In terms of endothelial function, after the TA was incubated with PPG, a significant increase in vasorelaxation occurred in the SHR (F_(1,78)_ = 15.5; *p* = 2.14 × 10^−4^), SHR + HFD (F_(1,58)_ = 18.4; *p* = 1.11 × 10^−4^), and SHR + HFD + GYY groups (F_(1,98)_ = 27.3; *p* = 1.35 × 10^−6^) (Figure 8A–C). However, AUC analyses revealed that in the TA, the significant antirelaxant effect of endogenous H_2_S was confirmed only in the SHR + HFD + GYY group (*p* < 0.05, Figure 8D). In the MA, the procontractile effect of H_2_S was confirmed in the control SHR group (*p* < 0.05), with PPG having no effect on either the SHR + HFD or SHR + HFD + GYY groups (Figure 9A–C), as confirmed by the AUC evaluation (*p* < 0.01, Figure 9D). With respect to endothelial function, PPG had no effect on the vasorelaxation response in the MA in any of the groups (Figure 10).

### 3.7. Total NOS Activity and Protein Expression Levels

**Figure 11:**
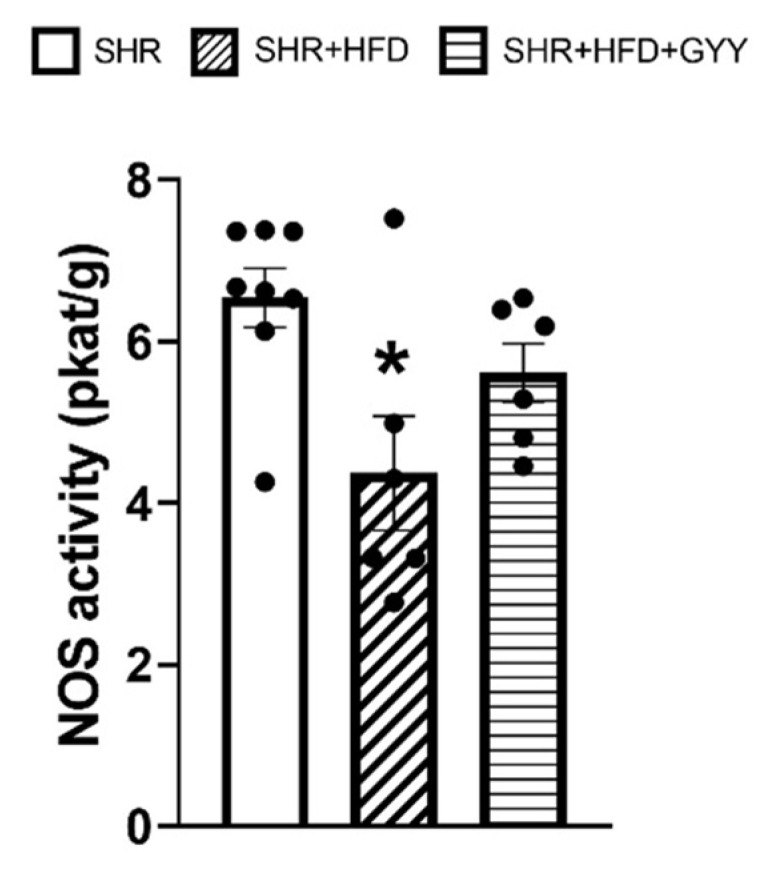
Total NOS activity in aortic tissue. Arteries were isolated from control rats (SHRs), SHRs fed the HFD (SHR + HFD), and SHRs fed the HFD and treated with GYY-4137 (SHR + HFD + GYY). The numbers of samples from the rats in each group were as follows: SHR *n* = 8, SHR + HFD *n* = 6, and SHR + HFD + GYY *n* = 6. The data are presented as the means ± S.E.Ms. The statistical analysis was performed by one-way ANOVA. * *p* < 0.05 compared with SHRs.

**Figure 12:**
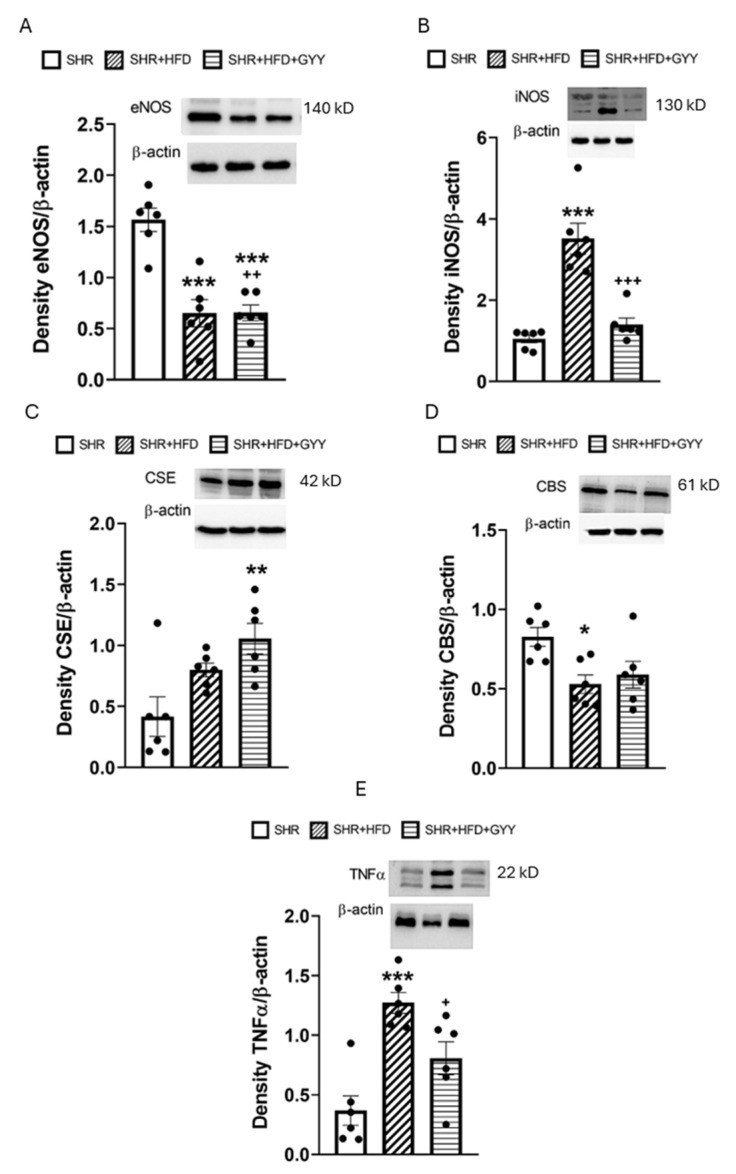
Protein expression of endothelial NOS (**A**), inducible NOS (**B**), the H_2_S-producing enzyme CSE (**C**), CBS (**D**), and TNFα (**E**) in aortic tissue. Arteries were isolated from control rats (SHRs), SHRs fed the HFD (SHR + HFD), and SHRs fed the HFD and treated with GYY-4137 (SHR + HFD + GYY). Endothelial NOS—eNOS; inducible NOS—iNOS; CSE—cystathionine γ-lyase; CBS—cystathionine β-synthase; TNFα—tumor necrosis factor alpha. The numbers of samples from rats in each group (for each protein) were as follows: SHR *n* = 6, SHR + HFD *n* = 6, and SHR + HFD + GYY *n* = 6. The data are presented as the means ± S.E.Ms. The statistical analysis was performed by one-way ANOVA. * *p* < 0.05, ** *p* < 0.01, and *** *p* < 0.001 compared with the SHR group; ^+^ *p* < 0.05, ^++^ *p* < 0.01, and ^+++^ *p* < 0.001 compared with the SHR + HFD group.

Total NO synthase (NOS) activity in the aorta was significantly lower in the high-fat diet group than in the control SHR group (*p* < 0.05) (Figure 11). This effect was confirmed by the decreased level of eNOS expression (*p* < 0.001) (Figure 12A). GYY-4137 administration increased the level of total NOS activity almost to the control level (Figure 11); however, the eNOS expression level decreased to an even greater extent than that in both the control and HFD groups (*p* < 0.001 and *p* < 0.01, respectively) (Figure 12A). On the other hand, chronic consumption of an HFD increased iNOS expression in the HFD group only (*p* < 0.001), and GYY-4137 administration decreased this value almost to the control level (Figure 12B).

Only simultaneous HFD and GYY-4137 administration increased the CSE protein level (*p* < 0.01), whereas the CBS protein level decreased after high-fat diet consumption (*p* < 0.05) (Figure 12C,D). In addition, the HFD significantly increased the TNFα expression level (*p* < 0.001); however, the administration of GYY-4137 partially reduced the levels of inflammatory markers (*p* < 0.05) (Figure 12E). Full scans of the entire original blots can be found in the Supplementary Materials.

## 4. Discussion

The first aim of our study was to characterize the dysfunction of the cardiovascular system, especially vascular function, induced by the long-term administration of a high-fat diet (HFD) to hypertensive rats. The second aim was to investigate the possible beneficial effects of the slow-releasing H_2_S donor GYY-4137 on treating the pathology in this model. We consider the finding that despite the insufficient effect of GYY-4137 on adiposity, dyslipidemia, and cardiac remodeling, the slow-releasing H_2_S donor triggered tissue specific beneficial vasoactive effects to be a new finding.

### 4.1. Effects of the High-Fat Diet

We confirmed that adiposity increased along with TAG levels in HFD-treated SHRs; however, unexpectedly, cholesterol levels decreased (Table 1). On the other hand, we previously showed that the administration of 10% fructose did not change the level of cholesterol [4] and Gaspárová et al. [12] confirmed an increase in the serum levels of total cholesterol and TAG after the administration of 60% fructose to SHR. Although HFD-induced dyslipidemia typically involves elevated cholesterol levels, the finding of decreased plasma cholesterol may be related to strain-specific differences in plasma lipid metabolism in SHR. Several authors have confirmed that the lower plasma cholesterol observed in hypertensive SHR was paralleled by specific differences in hepatic catalase and glutathione redox antioxidant enzyme activities and that increased cholesterol excretion associated with defects in molecular transport may also play a role [13,14]. A similar reduction in plasma cholesterol levels accompanied by an increase in plasma TAG levels was also confirmed in stroke-prone SHRs compared with control normotensive Wistar-Kyoto (WKYs), where a notable decrease in the cholesterol synthesis pathway was verified [13]. Furthermore, the effect of HFD on ectopic lipid deposition should also be considered. Hojná et al. [15] similarly to us found an increase in TAG and decrease in total cholesterol in plasma of SHR fed an HFD, which was associated with substantial ectopic accumulation of both cholesterol and TAG in the liver. We suggest that the presence of abnormal lipid metabolism in SHRs could persist or even be strengthened by increased lipid intake.

Although high-fat diets and high fructose intake are metabolically analogous, their effect in SHR may differ at the level of heart and BP regulation. Our previous results showed that fructose administration did not affect relative heart weight but led to an increase in BP due to, among others, increased fluid retention and stimulation of the aldosterone renin angiotensin system [16]. Shiou et al. [17] reported that after the consumption of an HFD, a significant decrease in BP occurred, along with cardiac dysfunction in both SHRs and WKYs, indicating lipotoxicity related to atrial and ventricular remodeling. In our study, excessive dietary fat intake did not affect BP but promoted the development of cardiac hypertrophy. Moreover, we also confirmed an increase in the protein expression of transforming growth factor-beta (TGF-β) and the amount of total collagen in the left ventricle (Table 2). Upon activation of TGF-β, fibroblasts differentiate into myofibroblasts, which are responsible for producing excessive extracellular matrix components, including collagen types I and III. This process is critical in tissue remodeling during fibrosis [18]. Concurrently, TGF-β upregulates the expression of tissue inhibitors of metalloproteinases, resulting in a net increase in ECM deposition and the progression of fibrosis [19]. Moreover, we found that the urea levels and urea-to-creatinine ratio in the HFD group were lower than those in the SHR group. The urea-to-creatinine ratio plays a role in monitoring and identifying acute kidney injury, and its decline could indicate potential intrinsic renal damage, which in turn increases the risk of developing hypertension and long-term cardiovascular damage [20,21]. Since we measured only the urea-to-creatinine ratio, further verification of HFD-associated kidney injury is needed. Nevertheless, the combination of an HFD and pre-existing hypertension appears to significantly affect the structure and function of the heart, emphasizing the need for a comprehensive understanding of all interactions to target therapeutic strategies.

Obesity and hypertension are comorbid conditions that act as independent risk factors for the onset of endothelial dysfunction. We found that the HFD did not affect endothelium-dependent vasorelaxation in the MA (Figure 2C). This result contrasts with our previous results, where improved endothelial function of MA associated with stimulation of the NO signaling pathway was confirmed in fructose-treated SHRs. This result also contrasts with the findings of Bosse et al. [22], who reported an improvement in acetylcholine-stimulated vasorelaxation associated with an increase in eNOS phosphorylation in the MA of SHRs. In contrast to our study, where the proportion of carbohydrates was 36% and that of fats was 45%, in that study, the proportion of carbohydrates was 20%, and that of the fats was 60%. Hence, different ratios of carbohydrate and fat intake could affect NO signaling and its possible role in compensatory mechanisms. Higher carbohydrate content could lead to insulin resistance, elevated glucose levels, and the generation of reactive oxygen species [23], which degrade NO and thereby impair vasodilation. Another explanation could be tissue specificity, since our results confirmed increased endothelium-dependent relaxation of the TA in the HFD group (Figure 2D). Nevertheless, the evaluation of the NO component by the AUC revealed that, in contrast to the other experimental groups, NO did not significantly participate in vasorelaxant responses in either artery in the HFD group (Figure 4D and Figure 6D). A similar result was observed in our previous study, where SHRs receiving a fructose diet for 8 weeks presented a reduced NO contribution to the vasorelaxant response in the TA [4]. Moreover, we also found that high-fat diet consumption decreased NOS activity and the expression of the endothelial NOS protein in aortic tissue (Figure 11 and Figure 12A), while the expression of inducible NOS (iNOS) was significantly increased. iNOS is expressed in response to stress, and elevated levels of iNOS protein expression, together with the increased expression of TNFα, trigger the inflammatory response [24].

The reduced eNOS protein production could be explained by the increased caveolin-1 expression that occurs in vascular tissue under HFD conditions [25]. In addition, eNOS can be regulated by post-translational modifications, particularly phosphorylation at specific serine and threonine residues, with kinases such as protein kinase B (Akt) and AMP-activated protein kinase being key regulators of this phosphorylation. AMP-activated protein kinase, as an energy sensor of the cell, and Akt, which can be activated by insulin signaling, represent a key link between the nutritional status of the organism and the functional activation of eNOS [26,27]. As confirmed by Tomada et al. [28], HFD intake led to a decrease in eNOS phosphorylation at the Ser1177 residue through the Akt pathway. Taken together, the activation of the NO signaling pathway was not responsible for the improved vasorelaxation of TA. Nevertheless, while NO, a key vasodilator produced by endothelial cells, is essential for maintaining vascular health and regulating blood pressure, other vasorelaxant agents, such as prostacyclin and the endothelium-derived hyperpolarizing factor (EDHF), which may compensate for the reduced NO availability, are also involved in the TA [29]. Thus, the activity of vasorelaxants other than NO, which may serve as a backup mechanism, could be responsible for the increase in vasorelaxation in the TA. In addition, NO may function as an important negative feedback regulator of the catalytic activity of its effector, soluble guanylate cyclase (sGC); hence, any reduction in the NO level may lead to an increase in the sensitivity of sGC to NO. A study by Jebelovszki et al. [30] confirmed that the administration of an HFD led to increased NO sensitivity in rat coronary arterioles because of sGC activation, which is also consistent with our findings, as we recorded an unchanged maximal response but increased sensitivity to acetylcholine.

Next, we assumed that H_2_S is the next relevant mediator, which would be consistent with it previously being identified as EDHF [31]. However, our results showed that the application of PPG, an inhibitor of the H_2_S-producing enzyme CSE, shifted the relaxation response curves to the left in the TAs of both the SHR and HFD groups (Figure 8A,B), indicating the antirelaxation effect of endogenously released H_2_S, although the evaluation of the AUC did not reveal any significant alterations in either the TAs or the MAs (Figure 8D and Figure 10D). Moreover, we found that the HFD had no significant effect on the CSE protein levels, and that CBS expression was slightly reduced. Similar results were reported in SHRs fed fructose, where endogenously produced H_2_S did not participate in endothelium-dependent relaxation [4], and in nonobese, hypertriglyceridemic rats, where endogenous H_2_S participated in the inhibition of endothelium-dependent vasorelaxation of the TA [32]. It seems that, in metabolic disorders, endogenously produced H_2_S generally has an antirelaxant effect rather than helping to maintain endothelial function.

With respect to contractility, we observed a significantly reduced contractile response to NA in both examined arteries following the consumption of an HFD. We observed a similar impairment of contractile abilities in both TA and MA in SHR after fructose administration. We proposed that the reduced contraction was not associated with stimulated NO/NOS participation, since decreased and unchanged NO involvement were observed in the TA and MA, respectively (Figure 3B,D and Figure 5B,D). Previously, Li et al. [33] confirmed that under an HFD regimen, vascular matrix remodeling, including thickening of the vessel wall, fibrosis, and apoptosis, was evident in the aortas of obese rats and was accompanied by increased arterial inflammation and oxidative stress. Moreover, Panchal et al. [34] observed reduced NA-induced contraction in the TA of HFD-fed rats, indicating smooth muscle dysfunction. Thus, we consider that the pathological conditions caused by an HFD might lead to a decreased contractile response through the induction of vascular remodeling of the arterial wall. TA, an elastic type of artery, is predisposed to increase the harmful remodeling induced by an HFD in SHRs. In our previous study, we confirmed a reduced vasocontractile response to NA in SHRs compared with that in Wistar rats, which was related to the fact that the component most involved in TA hypertrophy in adult SHRs was the extracellular matrix and not smooth muscle cells [35]. Regarding the MA, similarly to our findings, a diminished adrenergic contractile response induced by phenylephrine was observed by Bosse et al. [22] in SHRs fed a low-carbohydrate/high-fat diet. Although the authors consider this finding to be an improvement in vascular function, since the MA of SHRs is characterized by hypercontractility compared with that of controls, we assume that the observed compromised contractile function in both vessels is associated with a deteriorating (rather than beneficial) effect of the HFD on the contractile apparatus. Indeed, in our study, we detected increased expression of the iNOS and TNFα proteins in arterial tissue (Figure 12B,E), indicating the development of an inflammatory process, which could also lead to impaired contractility in both arteries. Moreover, we also confirmed a reduction in the plasmatic level of the adipokine chemerin, which has been proposed as a possible link between metabolic and vascular disorders and inflammation. Chemerin affects vascular function, which is mediated by the production of reactive oxygen species and redox signaling [36]. In addition, serum chemerin levels correlated with markers of inflammation, insulin resistance, and an unfavorable lipid profile, and have been proposed as a biomarker linking inflammation and cardiovascular diseases [37,38]. Similarly, Trovato et al. [39] showed that a high-fat Western diet could impair muscle metabolism, leading to muscle damage, which is associated with increased levels of inflammatory factors. Thus, we posit that contractile function was impaired by increased systemic inflammation under conditions of increased fat intake.

With respect to the sulfide signaling pathway, we found that, unlike in the TA, the HFD eliminated the pro-contractile effect of endogenous H_2_S observed in the control MA (Figure 6A,C). Although the anticontractile effect of endogenous sulfide signaling is usually considered compensatory and beneficial, in our experiments the loss of the pro-contractile effect of endogenously produced H_2_S contributed to the inhibition of contractile responses, at least in MA. On the other hand, after fructose intake, where we observed a similar impairment of contractile abilities in both TA and MA, this H_2_S action was not observed in any of the arteries and structural remodeling and inflammation were responsible for the impaired contractility.

Taken together, in SHRs, a high fat intake led to increased adiposity, elevated plasma triglyceride levels, and cardiac remodeling, as well as to generally decreased adrenergic contraction and reduced NO participation in vasoactive responses associated with decreased NOS activity, and reduced expression of the eNOS protein. The increased expression of iNOS and TNFα indicates that the initiation of the inflammatory process is likely responsible for the impairment of vascular function. Furthermore, comparison with previous experiments showed that while, with fructose intake, a backup mechanism for maintaining endothelial function was confirmed in both TA (NO-dependent) and MA (NO-independent) [4] with a high-fat diet, improved vasorelaxation capacity was confirmed only in TA (NO-independent). Although both dietary regimens resulted in impaired contractility of both arteries, likely due to inflammation and structural remodeling, the fibrotic changes in the heart, tissue-specific limitation of compensatory vasoactive capacities, and impaired contractility of MA associated with the action of endogenous H_2_S represent the original finding that high-fat consumption appears to impair the cardiovascular system of SHR more severely than fructose consumption.

### 4.2. Effects of the GYY-4137 Treatment

As a next step, we investigated the effect of the slow-releasing H_2_S donor GYY-4137 on HFD-induced metabolic and vasoactive changes. We found that body adiposity was not altered after GYY-4137 treatment, similar to that in fructose-fed SHR [4]. On the other hand, while GYY-4137 significantly reduced TAG levels in fructose-fed SHR, it had no effect on cholesterol and TAG levels in rats fed a high-fat diet (Table 1). The literature data suggest that H_2_S administration with GYY-4137 could reverse the chain of events leading to lipid accumulation in vitro and in vivo. Casili et al. [40] confirmed the suppressed lipid accumulation in adipocyte-like cells treated with GYY-4137 (6 mmol/L) in vitro. Zhao et al. (2020) [41] showed, in LDLr−/− mice treated with streptozotocin and an HFD, that 4 weeks of GYY-4137 treatment (133 μmol/L) attenuated tissue lipid deposition. Geng et al. [42] demonstrated that HFD consumption for 13 weeks resulted in downregulation of the CSE-H_2_S system in the adipose tissue of HFD-fed mice, while GYY-4137 treatment (200 μmol/kg/day) reduced lipolysis by inhibiting the phosphorylation of hormone-sensitive lipase. Based on these findings, it is surprising that the dose of GYY-4137 we used (266 μmol/L) had no significant effect on the lipid profile or other plasma markers in HFD-fed rats. On the other hand, Qabazard et al. [43] and Alshahwan et al. [44], who administered GYY-4137 at doses of 25 and 50 mg/kg for 28 days to streptozotocin-induced diabetic SD rats, showed that the efficacy of the donor is not proportional to its dose. Indeed, the regulation of adipose tissue lipolysis by H_2_S remains a matter of debate. The different metabolic and endocrine stages of pathological conditions may lead to the contradictory regulation of lipolysis, differences in the involvement of the sulfide signaling pathway, and inconsistent effects of H_2_S donors.

GYY-4137 treatment for 3 weeks profoundly reduced sBP compared with that in both HFD-fed and control SHRs (Figure 1). This finding is consistent with the finding of Li et al. [7], who reported that treatment with GYY-4137 decreased sBP in SHRs and that the reduction in sBP persisted for 14 days after the end of the treatment period. Another study in which SHRs were treated with GYY-4137 for 4 weeks reported reduced sBP, the inhibition of angiotensin II, and a reduced occurrence of myocardial fibrosis [9]. In our study, we did not observe a significant effect of GYY-4137 on the myocardial remodeling associated with HFD administration, although the amount of collagen in the left ventricle decreased. However, changes in cardiac trophicity may not be fully dependent on BP, as was confirmed during the ontogenesis of SHR [45]. Similarly, we recently showed that 3 weeks of treatment with GYY-4137 in fructose-fed SHRs resulted in a reduction in sBP but without an effect on cardiac parameters [4]. Although multiple mechanisms may be responsible for the GYY-4137-induced decrease in BP, the interaction with the renin-angiotensin-aldosterone system (RAAS) may play an important role. Despite SHRs being a normal-to-low renin and normal-to-low angiotensin/aldosterone model of hypertension [46], increased RAAS activity, along with elevated levels of angiotensin II, have been observed in HFD-fed animals [47]. Laggner et al. [48] showed that NaHS-generated H_2_S inhibited the activity of angiotensin-converting enzyme (ACE) in endothelial cells. In our previous study, we confirmed that an in vivo bolus administration of the ACE inhibitor captopril reduced the H_2_S donor-induced decrease in BP, suggesting that captopril disabled and masked the inhibitory effect of H_2_S on the RAAS [49]. We posit that the inhibition of the RAAS could be responsible for the decrease in sBP induced by the administration of GYY-4137.

Treatment with GYY-4137 increased the endothelium-dependent relaxation of both arteries, although some differences were observed between the TA and MA. A similarly increased vasorelaxation of the TA was found in both the HFD and GYY groups; however, only the GYY-4137 treatment increased endothelium-dependent vasorelaxation in the MA compared with both the control SHR and the HFD-treated rats (Figure 2C,D). Furthermore, we observed that NO participation in vasorelaxant responses in both the TA and MA was restored after the administration of GYY-4137, although the anti-relaxation action of endogenous H_2_S was confirmed in TA. On the other hand, after fructose intake, the endothelium-dependent relaxation of both arteries was similarly increased after treatment with GYY-4137; while in MA this effect was mediated by the increased participation of NO, in TA the pro-relaxant action of endogenous H_2_S was responsible. These findings suggest that GYY-4137 administration leads to activation of one of the pathways (nitrate or sulfide), which could be tissue-specific and influenced by the origin of the metabolic disorder. We hypothesize that GYY, which acts as an H_2_S donor, could, in high-fat-fed rats, stimulate the production of endogenous H_2_S, which was confirmed by the increased expression of CSE in aortic tissue. An increased supply of H_2_S could subsequently sulfhydrate eNOS [50] and restart its activity. This result was also supported by the finding that, unlike in the HFD group, NOS activity did not remain reduced after GYY administration, and a trend toward increasing NOS activity was noted; thus, no difference was observed compared with the control group. Surprisingly, the decrease in endothelial NOS expression observed after HFD consumption was further exacerbated by GYY-4137 administration. We assume that a negative feedback mechanism may have been behind this effect. eNOS expression is known to be activated by the proinflammatory factor NFκB as part of a negative feedback loop, such that increases in NO levels (and NOS activity) that repress NF-κB might lead to decreased levels of eNOS and vice versa [51]. Taken together, these findings indicate that GYY-4137 administration improved endothelial function, which was likely related to the restoration of the NO signaling pathway, and endogenously produced H_2_S did not directly contribute to this effect, as it was either not involved (MA) or was involved in the antirelaxation (TA) response. Nevertheless, it appears that the administration of exogenous H_2_S donor helps maintain the balance between nitrous and sulfide signaling, which can be disrupted due to modifications in both lipid and saccharide metabolism.

HFD-induced impairments in contraction were only partially ameliorated by GYY-4137 treatment in the TA, highlighting the tissue-specific effects of this donor, which could also be related to the different biomechanical properties of the MA and TA or smooth muscle phenotypic differences. Soares et al. [52] confirmed greater smooth muscle cell damage in the small MA than in the TA in the obese mice fed an HFD, which was associated with the higher expression of genes required for maintaining contractile capacity in the TA than in the MA and increased collagen deposition in the MA but not in the TA. Moreover, phenotypic differences between aortic and mesenteric perivascular adipose tissue (PVAT) could also take place. Aortic PVAT generates far fewer proinflammatory cytokines and thus could be more resistant to diet-induced inflammation. Mesenteric PVAT is more sensitive to the high-fat diet challenge, where the adipose “browning” genes are dramatically down-regulated [53]. With respect to the endogenous nitrous and sulfide signaling pathways, both NO and H_2_S likely did not contribute to the action of GYY-4137 in the TA; compared with those in the HFD group, the proportion of NO or H_2_S involvement in the contractile response did not change (Figure 3D and Figure 7D). We posit that the improvement in the contractile response in the GYY group could be related to the anti-inflammatory effect of the H_2_S donor. Indeed, H_2_S has notable anti-inflammatory effects, such as reducing the levels of proinflammatory cytokines, chemokines, and enzymes by inhibiting the activation of NFκB [54]. The anti-inflammatory role of H_2_S is further supported by our finding that the GYY-4137 treatment attenuated HFD-induced increases in iNOS and TNFα levels (Figure 12B,D), which is consistent with the finding that GYY-4137 has anti-inflammatory activity in disease states. Li et al. [55] showed that in LPS-stimulated human synoviocytes, GYY-4137 decreased TNFα and IL-6 production, reduced iNOS levels, and inhibited NFκB activation. Similarly, GYY-4137 significantly reduced the serum levels of TNFα and interleukin-6 (IL-6), as well as iNOS expression, in mice with sepsis [56].

### 4.3. Study Limitations

After both the high-fat diet and GYY-4137 treatment, we observed tissue-specific vascular changes depending on the type of artery, elastic vs. muscular. For a deeper analysis, future research will need to focus on the detailed specification of signaling pathways and regulatory mechanisms using in vivo (e.g., pulse wave velocity measurement) and in vitro (e.g., Western blot, real-time PCR analysis) methods. Also, the isolation and cultivation of endothelial cells and smooth muscle cells from specific types of arteries will allow for the study of their individual properties and responses to different stimuli. Regarding the effects of GYY-4137, although GYY-4137 treatment generally reduced BP, the limiting factor could be the insufficient effect of GYY-4137 on adiposity and dyslipidemia, which may be related to the method (single dose) and duration of administration. Therefore, whether GYY-4137 can improve long-term outcomes in disorders associated with HFD should be assessed in the future. The metabolic changes induced by a high fat intake are complex and multifactorial; therefore, long-term studies should be considered when evaluating its potential therapeutic effects. Finally, understanding the physiological roles of H_2_S compared to NO, as well as the stability of H_2_S donors, poses a challenge for effective treatment.

## 5. Conclusions

In conclusion, despite the insufficient effect of GYY-4137 on adiposity, dyslipidemia, and cardiac remodeling, the slow-releasing H_2_S donor triggered beneficial vasoactive effects: a decreased BP, partially improved MA relaxation and TA contraction, which was associated with restored NO signaling, and decreased expression of the iNOS and TNFα proteins. On the other hand, under high-fat diet conditions, endogenous H_2_S did not participate in the improvement in vascular function. Our results suggest that slow-releasing H_2_S donors could partially ameliorate metabolic changes and trigger beneficial vasoactive effects associated with the recovery of NO signaling and the suppression of inflammation.

## Figures and Tables

**Table 1:** General parameters of the experimental animals.

Parameters	SHR	*n*	SHR + HFD	*n*	SHR + HFD + GYY	*n*
BW (g)	282.1 ± 13.3	8	332.4 ± 6.3 ***	7	327.3 ± 7.3 **	8
HW (g)	1.047 ± 0.05	8	1.335 ± 0.03 ***	7	1.372 ± 0.05 ***	8
RTW (g)	2.07 ± 0.24	8	3.40 ± 0.20 **	7	3.44 ± 0.24 **	8
TL (mm)	34.42 ± 0.27	8	37.51 ± 0.9 **	7	37.05 ± 0.55 **	8
RTW/BW (mg/g)	7.21 ± 0.6	8	10.21 ± 0.46 **	7	10.48 ± 0.55 ***	8
RTW/TL (mg/mm)	60.38 ± 7.14	8	90.59 ± 4.38 **	7	93.24 ± 6.71 **	8
GLU (mmol/L)	8.26 ± 0.4	8	8.68 ± 0.4	7	8.09 ± 0.3	8
TAG (mmol/L)	1.20 ± 0.07	8	2.42 ± 0.20 ***	7	2.07 ± 0.24 **	8
CHOL (mmol/L)	2.67 ± 0.19	8	2.32 ± 0.07 *	7	2.53 ± 0.10	8
Urea (mmol/L)	7.86 ± 0.11	8	7.09 ± 0.13 **	7	7.26 ± 0.26	8
CRE (umol/L)	23.57 ± 3.40	8	33.14 ± 4.38	7	34 ± 11.4	8
Urea/CRE	361.72 ± 32.76	8	237.27 ± 31.90 *	7	305.05 ± 031.06	8
Chemerin (ng/mL)	158.25 ± 3.28	6	220.94 ± 7.66 *	7	161.31 ± 5.91 ^+^	8
Food intake (g/day)	20.87 ± 0.45	8	15.56 ± 0.15 ***	7	14.90 ± 0.16 ***^+^	8

Abbreviations: *n*—number of rats; SHR—spontaneously hypertensive rats; SHR + HFD—spontaneously hypertensive rats receiving a high-fat diet; SHR + HFD + GYY—spontaneously hypertensive rats receiving an HFD and the H_2_S donor GYY-4137; BW—body weight; RTW—retroperitoneal adipose tissue weight; TL—tibia length; RTW/BW—ratio of retroperitoneal adipose tissue weight to body weight; RTW/TL—ratio of retroperitoneal adipose tissue weight to tibia length; GLU—glucose; TAG—triacylglycerol; CHOL—cholesterol; CRE—creatinine; Urea/CRE—ratio of urea to creatinine. The values are presented as the means ± S.E.Ms; * *p* < 0.05, ** *p* < 0.01 and *** *p* < 0.001 compared with the SHR group, ^+^ *p* < 0.05 compared with the SHR + HFD group. Statistical analyses were performed by one-way ANOVA with the Bonferroni post hoc correction.

**Table 2:** Cardiac parameters of the experimental animals.

Parameters	SHR	*n*	SHR + HFD	*n*	SHR + HFD + GYY	*n*
HW (g)	1.047 ± 0.05	8	1.335 ± 0.03 ***	7	1.372 ± 0.05 ***	8
HW/BW (mg/g)	3.72 ± 0.78	8	4.02 ± 0.07 *	7	4.19 ± 0.13 *	8
HW/TL (mg/mm)	30.43 ± 1.52	8	35.69 ± 0.85 *	7	37.08 ± 1.44 **	8
TGF-β (Density TGF-β/ β-actin)	0.11 ± 0.01	6	2.45 ± 0.16 ***	6	1.41 ± 0.17 ^+++^	6
Collagen (μg/mg)	6.65 ± 0.77	6	9.14 ± 0.57 *	6	6.79 ± 0.46 *	6

Abbreviations: *n*—number of rats; SHR—spontaneously hypertensive rats; SHR + HFD—spontaneously hypertensive rats receiving a high-fat diet; SHR + HFD + GYY—spontaneously hypertensive rats receiving an HFD and the H_2_S donor GYY-4137; HW—heart weight; HW/BW—ratio of heart weight to body weight; HW/TL—ratio of heart weight to tibia length; TGF-β—protein expression of transforming growth factor-β in the left ventricle; Collagen—the amount of collagen in the left ventricle. The values are presented as the means ± S.E.Ms.; * *p* < 0.05, ** *p* < 0.01 and *** *p* < 0.001 compared with the SHR group, ^+++^ *p* < 0.01 compared with the SHR + HFD group. Statistical analyses were performed by one-way ANOVA with the Bonferroni post hoc correction.

**Table 3:** Characterization of noradrenaline-induced contraction and acetylcholine-induced relaxation in the thoracic aorta.

	Thoracic Aorta					
	SHR	*n*	SHR + HFD	*n*	SHR + HFD + GYY	*n*
NA_max_ (g)	0.58 ± 0.03	7	0.37 ± 0.07 ***	7	0.44 ± 0.03 *^+^	8
NA EC_50_ (−log mol/L)	8.39 ± 0.25	7	9.08 ± 0.26	7	9.53 ± 0.07 ***	8
Ach_max_ (%)	78.59 ± 4.75	7	76.95 ± 5.54	6	78.74 ± 5.79	7
Ach EC_50_ (−log mol/L)	8.70 ± 0.19	7	9.30 ± 0.17 *	6	9.37 ± 0.09 **	7

Abbreviations: *n*—number of rats; SHR—spontaneously hypertensive rats; SHR + HFD—spontaneously hypertensive rats receiving the high-fat diet; SHR + HFD + GYY—spontaneously hypertensive rats receiving the HFD and H_2_S donor GYY-4137; NA—noradrenaline; NAmax—maximum noradrenaline-induced contraction; NA EC50—the negative logarithm of the NA molar concentration inducing the half-maximal response; Ach—acetylcholine; Achmax—maximum acetylcholine-induced relaxation; Ach EC50—the negative logarithm of the Ach molar concentration inducing the half-maximal response. Values are presented as the means ± S.E.Ms. * *p* < 0.05, ** *p* < 0.01, and *** *p* < 0.001 compared with the SHR group; ^+^ *p* < 0.05 compared with the SHR + HFD group. The statistical analysis was performed by one-way or two-way ANOVA with the Bonferroni post hoc correction.

**Table 4:** Characterization of noradrenaline-induced contraction and acetylcholine-induced relaxation in the mesenteric artery.

	Mesenteric Artery					
	SHR	*n*	SHR + HFD	*n*	SHR + HFD + GYY	*n*
NA_max_ (g)	1.18 ± 0.06	7	0.78 ± 0.06 ***	6	0.74 ± 0.04 ***	7
NA EC_50_ (−log mol/L)	7.43 ± 0.07	7	7.41 ± 0.13	6	7.42 ± 0.10	7
Ach_max_ (%)	43.97 ± 3.97	7	40.68 ± 2.80	6	47.72 ± 3.12 *^+^	6
Ach EC_50_ (−log mol/L)	7.56 ± 0.18	7	7.74 ± 0.29	6	8.18 ± 0.43	6

Abbreviations: *n*—number of rats; SHR—spontaneously hypertensive rats; SHR + HFD—spontaneously hypertensive rats receiving the high-fat diet; SHR + HFD + GYY—spontaneously hypertensive rats receiving the HFD and H_2_S donor GYY-4137; NA—noradrenaline; NAmax—maximum noradrenaline-induced contraction; NA EC50—the negative logarithm of the NA molar concentration inducing the half-maximal response; Ach—acetylcholine; Achmax—maximum acetylcholine-induced relaxation; Ach EC50—the negative logarithm of the Ach molar concentration inducing the half-maximal response. Values are presented as means ± S.E.Ms. * *p* < 0.05 and *** *p* < 0.001 compared with the SHR group; ^+^ *p* < 0.05 compared with the SHR + HFD group. The statistical analysis was performed by one-way or two-way ANOVA with the Bonferroni post hoc correction.

## Data Availability

The raw data supporting the conclusions of this study are available from the corresponding author upon request, without undue reservation. Our work has been made available as preprint: Aydemir B.G., Berenyiova A., Cebova M. et al. The Effect of High Fat Diet and H2S Donor GYY-4137 on Vascular Function in Spontaneously Hypertensive Rats, 17 April 2025, PREPRINT (Version 1) available at Research Square [https://doi.org/10.21203/rs.3.rs-6030564/v1].

## Supplementary Materials

The following supporting information can be downloaded at: https://www.mdpi.com/article/10.3390/pathophysiology32020027/s1, Full scans of the entire original blots for eNOS, iNOS, CSE, CBS and TNFα can be found in the Supplementary Materials.

## Abbreviations

The following abbreviations are used in this manuscript:

Ach	acetylcholine
AUC	areas under the curve
BW	body weight
CaCl_2_	calcium chloride
CaNa_2_EDTA	edetate calcium disodium
CBS	cystathionine β
synthase	
CHOL	cholesterol
CO_2_	carbon dioxide
CRE	creatine
CSE	cystathionine γ
lyase	
DMSO	dimethyl sulfoxide
DMAB	dimethylaminobenzaldehyde
ECL	enhanced chemiluminescence
EDHF	endothelium
derived hyperpolarizing factor	
eNOS	endothelial nitric oxide synthase
GLU	glucose
H_2_S	hydrogen sulfide
HFD	high
fat diet	
HW	heart weight
HYP	hydroxyproline
IL	6
interleukin	6
iNOS	inducible nitric oxide synthase
KCl	potassium chloride
KH_2_PO_4_	monopotassium phosphate
LN	N(G)
nitro	L
arginine methyl ester	
MA	mesenteric artery
MgSO_4x_7H_2_O	magnesium sulfate heptahydrate
NA	noradrenaline
Na_2_S	sodium sulfide
NaCl	sodium chloride
NaHCO_3_	sodium hydrogen carbonate
NaHS	sodium hydrosulfide
NaOH	sodium hydroxide
NO	nitric oxide
NOS	nitric oxide synthase
O_2_	oxygen
PPG	DL
propargylglycine	
PVAT	perivascular adipose tissue
RAAS	renin
angiotensin	aldosterone system
RTW	retroperitoneal adipose tissue weight
sBP	systolic blood pressure
sGC	soluble guanylate cyclase
SHR	spontaneously hypertensive rats
TA	thoraric aorta
TAG	triacylglycerol
TGF	β
transforming growth factor	beta
TL	the length of the tibia
TNFα	tumor necrosis factor alpha
WKY	Wistar
Kyoto	
