# Supplementary material for: A Slow Hydrogen Sulfide Donor GYY-4137 Partially Improves Vascular Function in Spontaneously Hypertensive Rats Fed a High-Fat Diet
Source: Pathophysiology. 2025 Jun 18;32(2):27. doi: 10.3390/pathophysiology32020027 (PMC12196443; doi:10.3390/pathophysiology32020027)
Supplement: Supplementary file 1 [file pathophysiology-32-00027-s001.zip › pathophysiology-3658123-supplementary.pdf]

A full scan of the entire original gel of blots for eNOS, iNOS, CSE, CBS and TNFα. On the original blots, there is an indication of which band we evaluated (iNOS, eNOS and TNF-alpha presented more than one band, indicate the band analyzed), moreover the first three columns in each blot belong to the groups in the order: SHR, SHR+HFD, SHR+HFD+GYY. For evaluation of samples, we used Protein standard. According to the molecular weight of the manufacturer of the antibody followed by reference band we evaluated individual samples. Protein concentration used for WB – 20µg/well. Since we re-probe the membrane for more proteins, we decided to use 12% gel instead 7.5%

eNOS 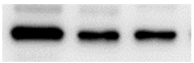 140 kDa

β-actin 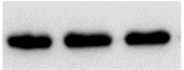 42 kDa

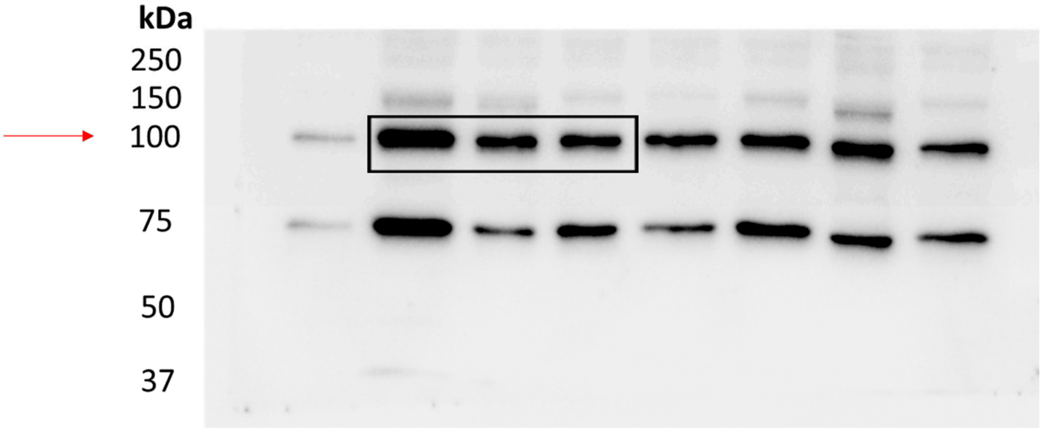

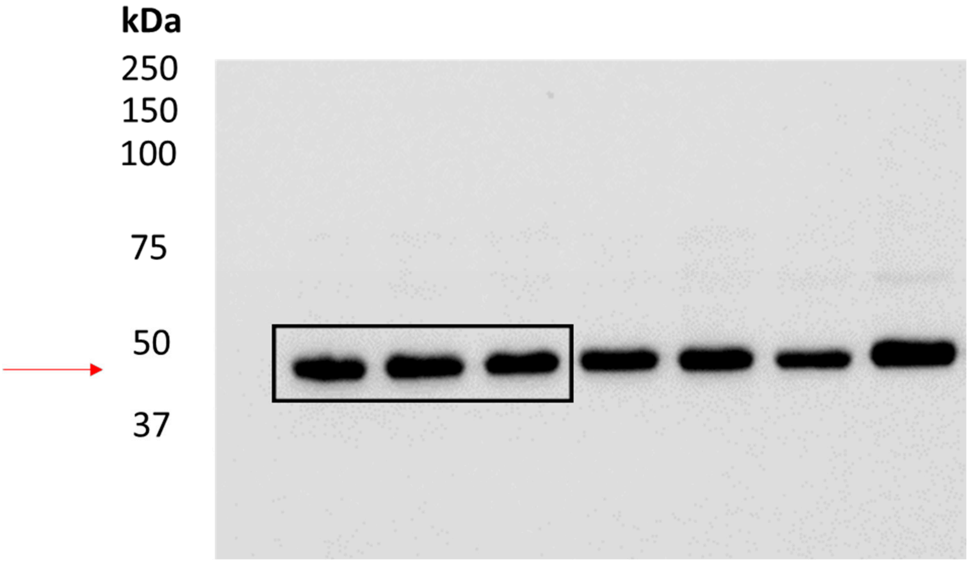

iNOS

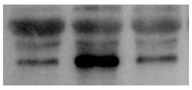

130 kDa

β-actin

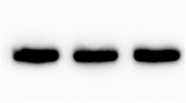

42 kDa

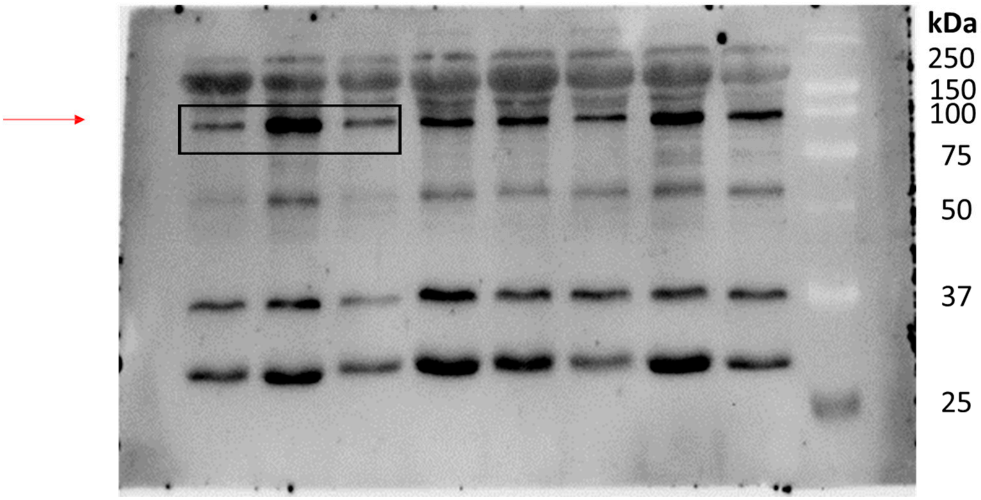

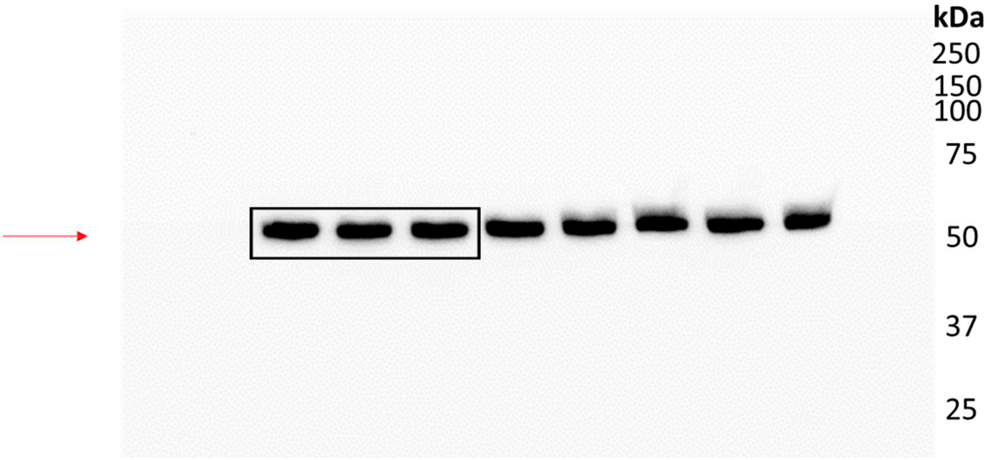

CSE 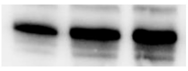 42 kDa

β-actin 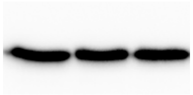 42 kDa

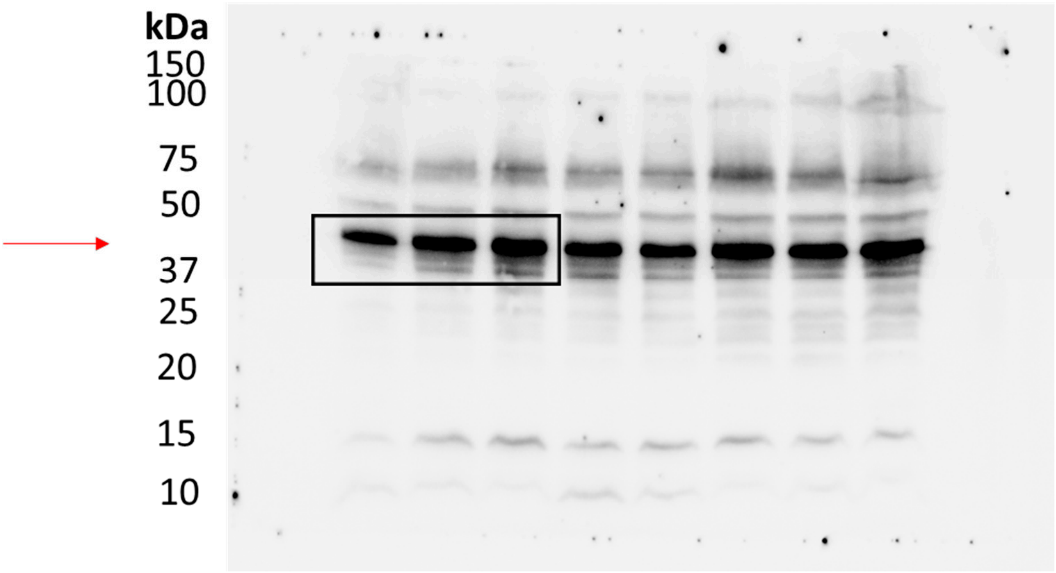

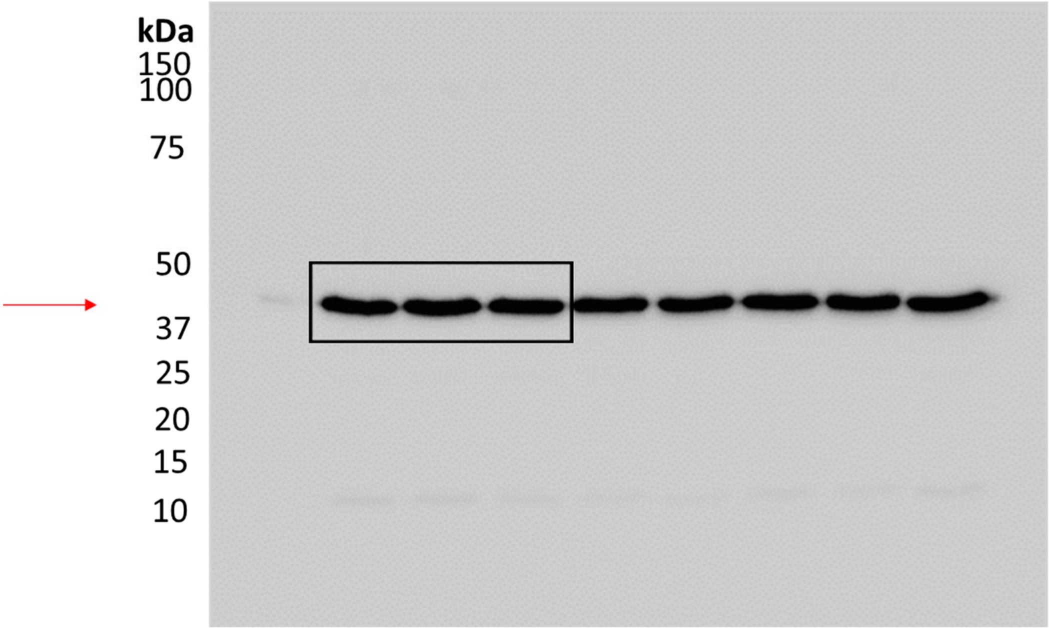

TNF-α 22 kDa

β-actin 42 kDa

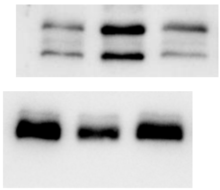

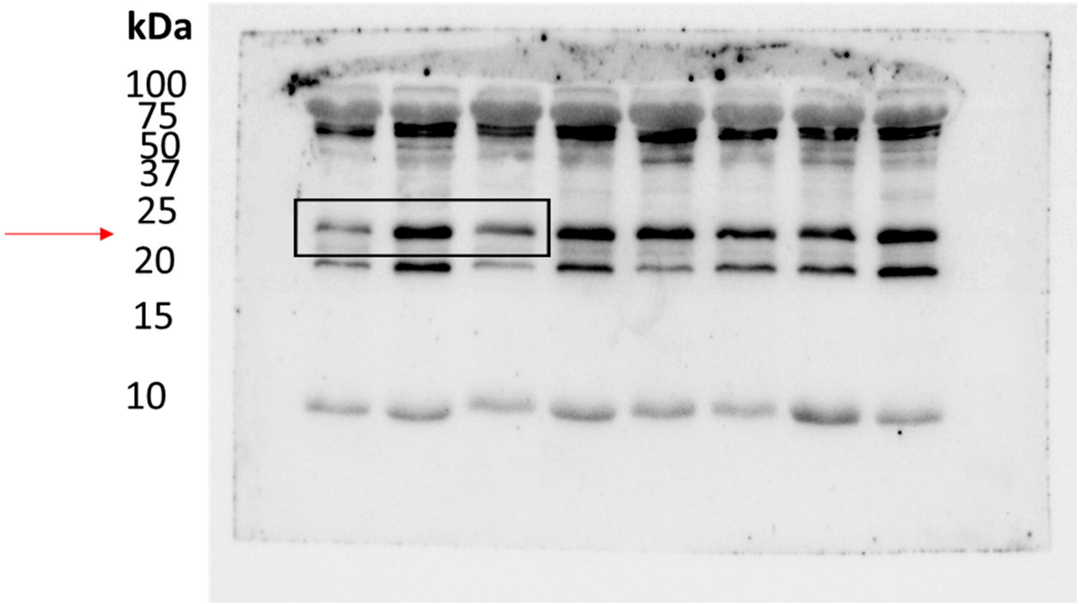

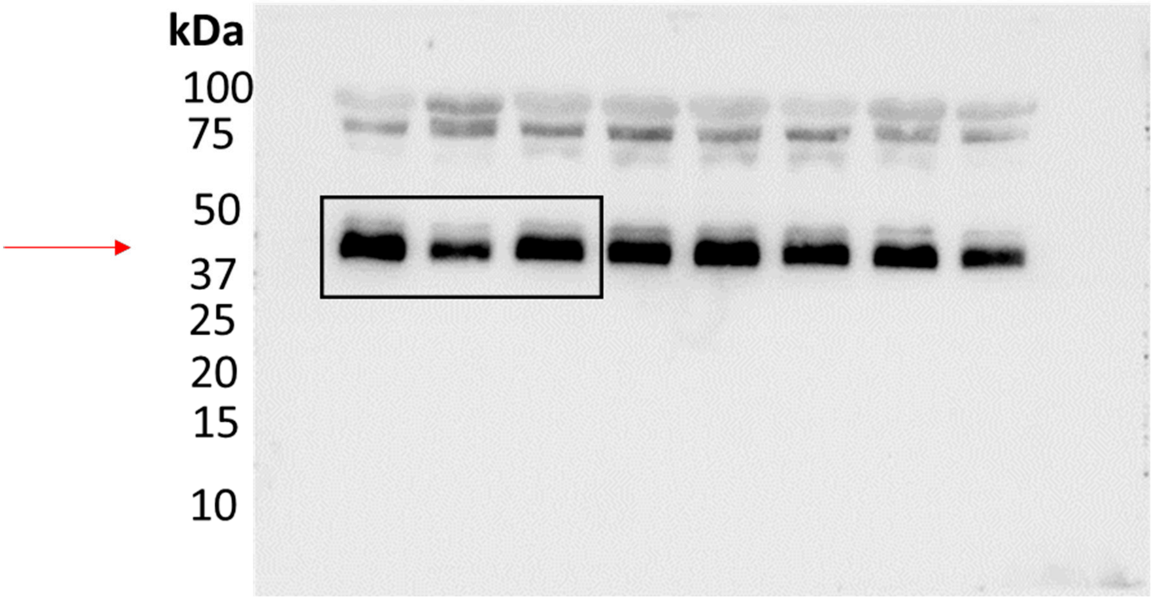

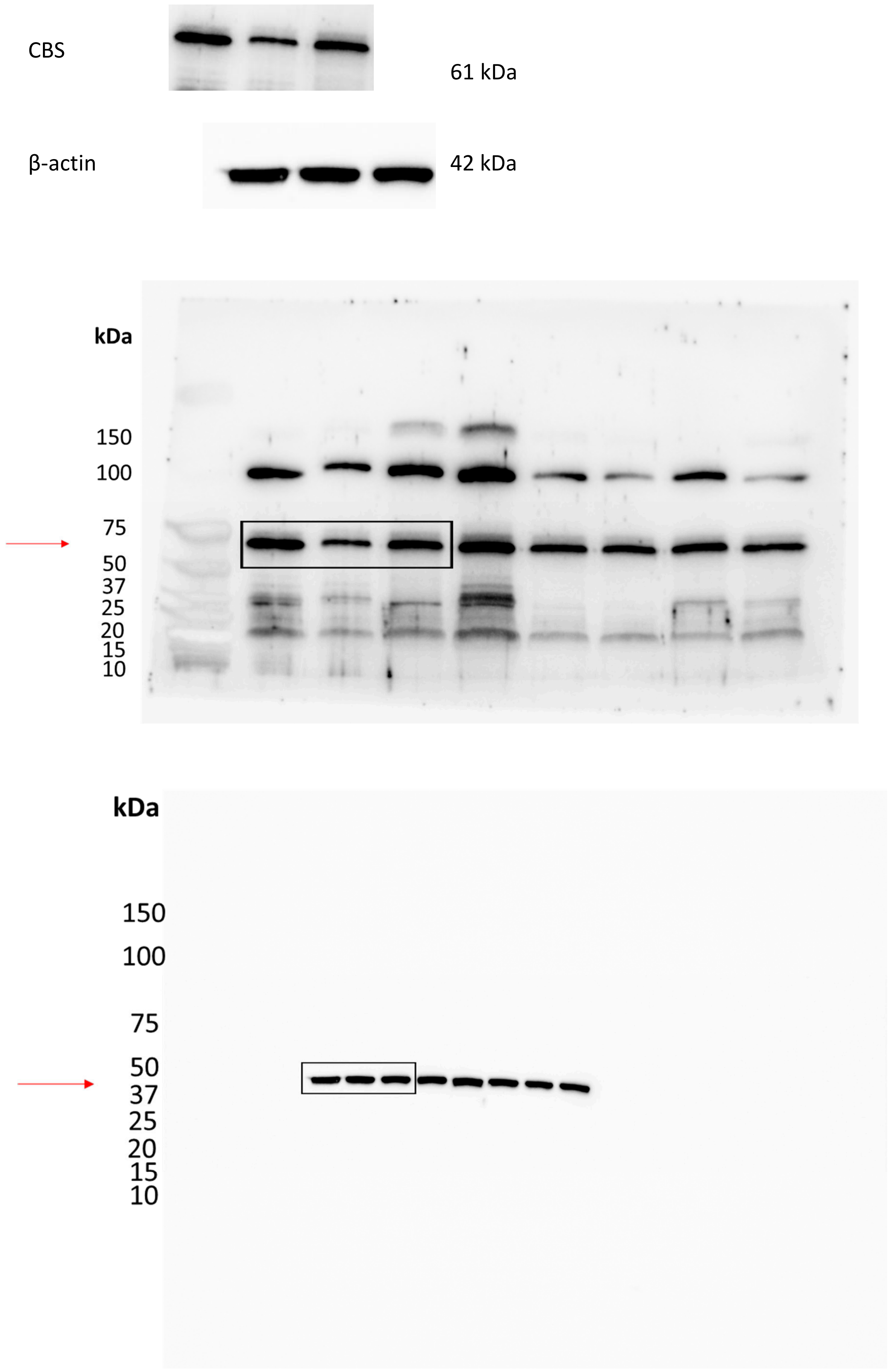
CBS
61 kDa
β-actin
42 kDa
kDa
150
100
75
50
37
25
20
15
10
kDa
150
100
75
50
37
25
20
15
10
